# Biphenyls in Clusiaceae: Isolation, structure diversity, synthesis and bioactivity

**DOI:** 10.3389/fchem.2022.987009

**Published:** 2022-12-01

**Authors:** Youyi Wang, Qing Huang, Li Zhang, Changwu Zheng, Hongxi Xu

**Affiliations:** ^1^ School of Pharmacy, Shanghai University of Traditional Chinese Medicine, Shanghai, China; ^2^ Shuguang Hospital, Shanghai University of Traditional Chinese Medicine, Shanghai, China

**Keywords:** *Garcinia* genus, biphenyl, prenyl, cytotoxicity, antibacterial activity

## Abstract

Clusiaceae plants contain a wide range of biologically active metabolites that have gotten a lot of interest in recent decades. The chemical compositions of these plants have been demonstrated to have positive effects on a variety of ailments. The species has been studied for over 70 years, and many bioactive compounds with antioxidant, anti-proliferative, and anti-inflammatory properties have been identified, including xanthones, polycyclic polyprenylated acylphloroglucinols (PPAPs), benzophenones, and biphenyls. Prenylated side chains have been discovered in many of these bioactive substances. To date, there have been numerous studies on PPAPs and xanthones, while no comprehensive review article on biphenyls from Clusiaceae has been published. The unique chemical architectures and growing biological importance of biphenyl compounds have triggered a flurry of research and interest in their isolation, biological evaluation, and mechanistic studies. In particular, the FDA-approved drugs such as sonidegib, tazemetostat, daclatasvir, sacubitril and trifarotene are closely related to their biphenyl-containing moiety. In this review, we summarize the progress and development in the chemistry and biological activity of biphenyls in Clusiaceae, providing an in-depth discussion of their structural diversity and medicinal potential. We also present a preliminary discussion of the biological effects with or without prenyl groups on the biphenyls.

## 1 Introduction

Clusiaceae family is extensively used in traditional medicine for treating a number of diseases which include cancer, inflammation, and infection (Acuna et al., 2009). There are many species belonging to the Clusiaceae family, such as *Clusia* L., *Garcinia* L., *Pentadesma* Sabine and so on. Among them, *Garcinia* is a representative species comprising of about 400 recognized plants and is extensively dispersed throughout tropical and subtropical Asia, Africa, and America (2022). The chemical compositions of these plants contain a variety of valuable natural products with associated beneficial and healing properties such as anti-tumor (Fan et al., 2015), anti-obesity (Hasegawa, 2001; Kim et al., 2004), antibacterial (Negi et al., 2008; Auranwiwat et al., 2014), antioxidant (Farombi et al., 2013), antimalarial (Elfita et al., 2009), and so on (Grossman and Yang, 2020; Spontoni do Espirito Santo et al., 2020; Kalita et al., 2021). As a result, a considerable bunch of studies have been conducted to evaluate all types of phytochemical compositions from Clusiaceae, including polycyclic polyprenylated acylphloroglucinols (PPAPs) (Tian et al., 2016; Chen et al., 2020), xanthones (Nilar and Harrison, 2002; Rukachaisirikul et al., 2003b; Elfita et al., 2009), benzophenones (Elya et al., 2006), flavonoids (Nguyen et al., 2021), biphenyls (Auranwiwat et al., 2021) and others (Ly Dieu et al., 2012; Jiang et al., 2014).

**FIGURE 1:**
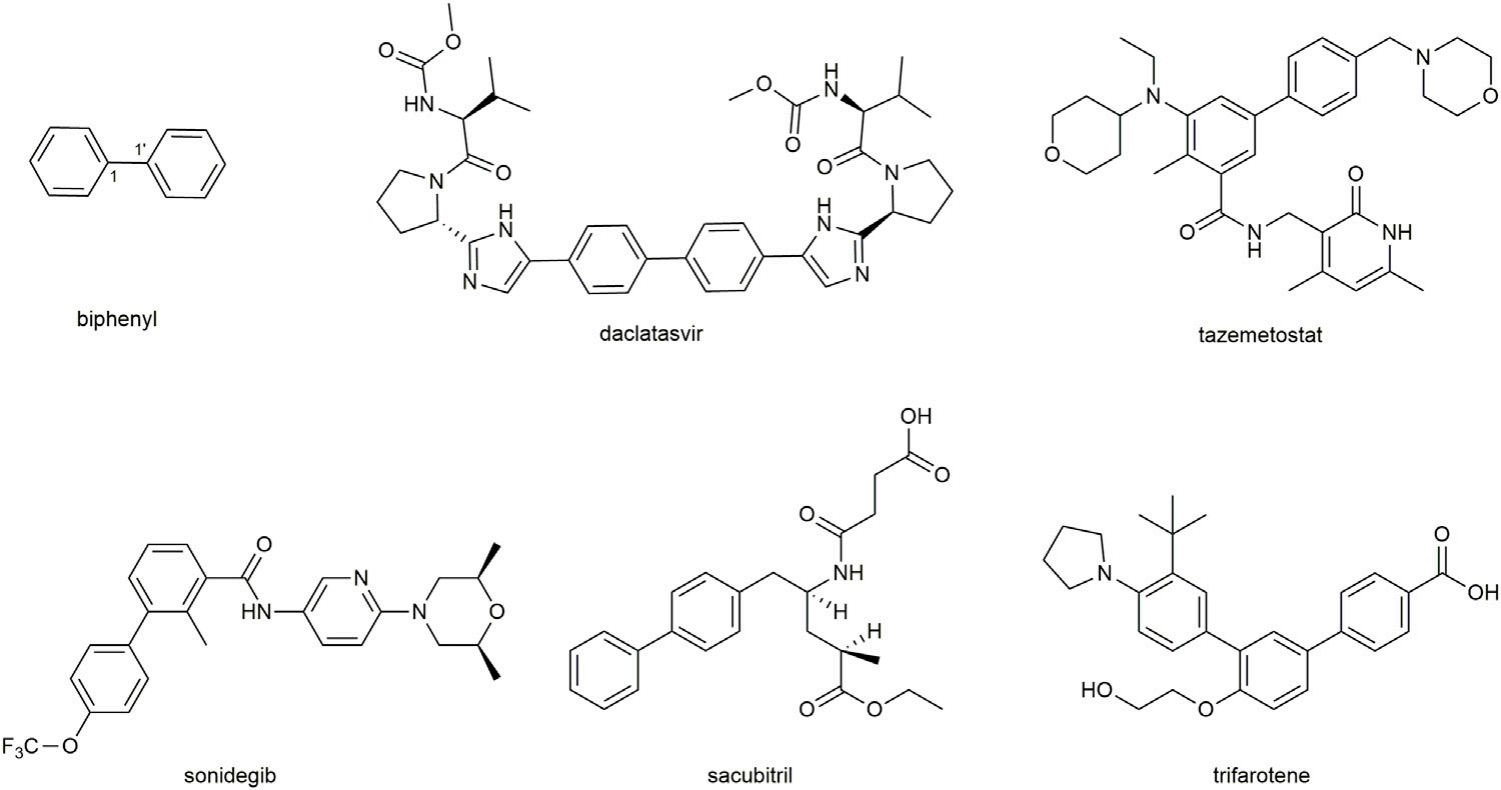
Basic structure of biphenyl and five FDA-approved drugs containing biphenyl structure from 2015-June 2020.

**FIGURE 2:**
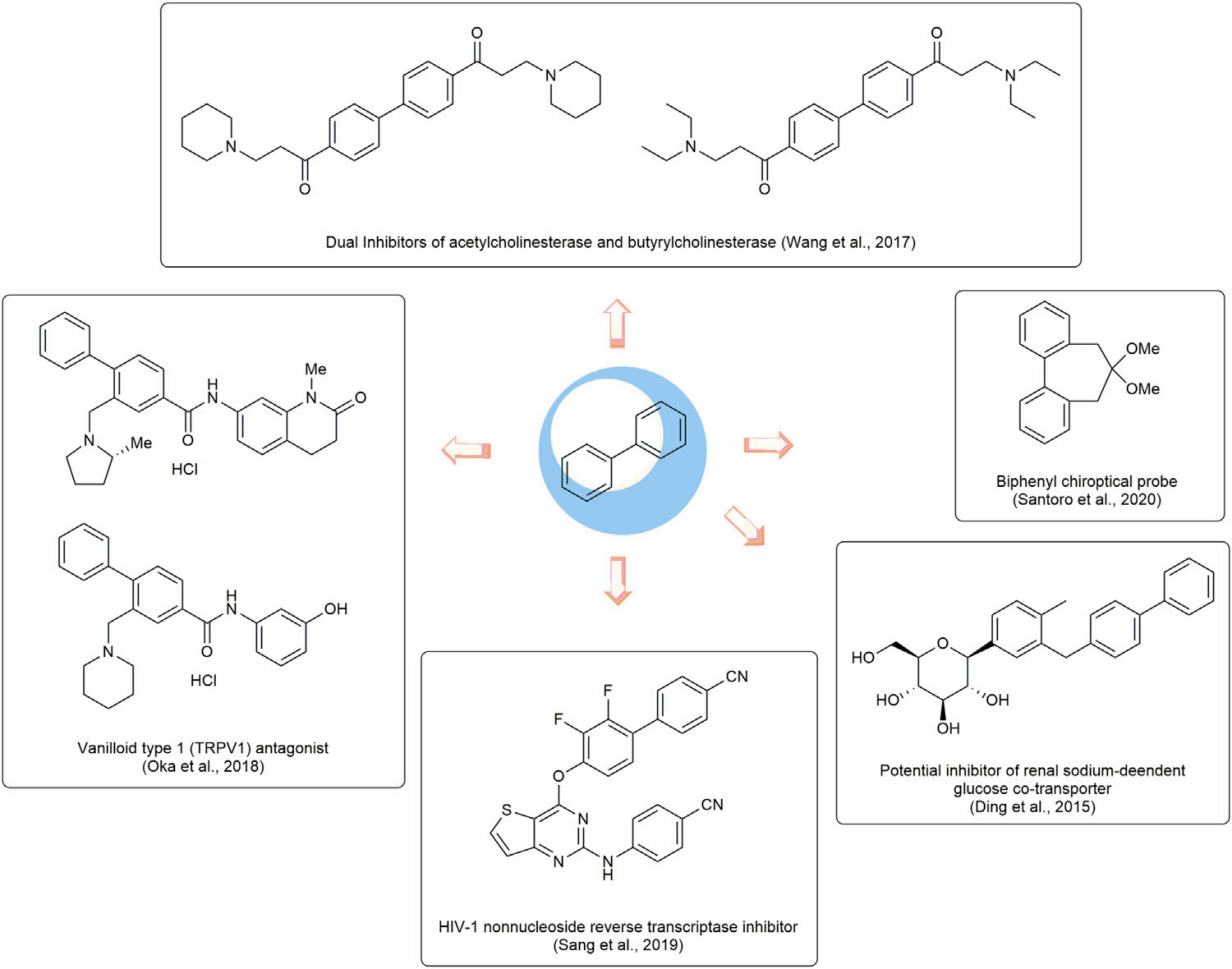
Development of bioactive compounds based on biphenyl.

Biphenyls are odorant and white crystals typically consisting of two adjacent benzene rings connected by a C1-C1’ bond (Kwong et al., 2017) (Figure 1). The biphenyl is normally used as an advantaged and functional moiety in the field of drug design and the process of medicinal advancement (Lu et al., 2015; Anusha et al., 2016; Tarade et al., 2017; Delgado et al., 2019). They have the potentials to be developed as prospecting therapeutic agents for a range of diseases (Chen et al., 2022), such as the dual inhibitors of acetylcholinesterase and butyrylcholinesterase (Wang et al., 2017) for Alzheimer’s disease (Inuzuka et al., 2022), the transient receptor potential vanilloid type 1 (TRPV1) antagonist (Oka et al., 2018), the human immunodeficiency virus -1 (HIV-1) nonnucleoside reverse transcriptase inhibitor (Sang et al., 2019). In addition, they could also be applied as auxiliary parts to enhance the biological properties (Ding et al., 2015) or act as chiroptical probes (Santoro et al., 2020). It is noted that some FDA-approved drugs, such as sonidegib, tazemetostat, daclatasvir, sacubitril, and trifarotene (Figure 2), have been developed in recent years with biphenyl as the core structure (Bhutani et al., 2021).

So far, Clusiaceae has yielded a variety of critical compounds with remarkable activity, such as α-mangostin (Gutierrez-Orozco and Failla, 2013), gambogic acid (Banik et al., 2018), oblongifolin C (Li et al., 2018) and so on. The majority of these key compounds belong to the structurally unique PPAPs and xanthones, which are the two principal metabolites (Chantarasriwong et al., 2010; Li et al., 2016; Xu et al., 2020). Biphenyls are secondary metabolites regularly yielded especially from individual species of the *Garcinia* genus (Chen et al., 2019). In addition, biphenyl can be classified as a phytoalexin that is produced in response to pathogen assault (Zhou et al., 2016) and it can be extracted from a wide range of plants with related bioactive ability (Teixeira et al., 2006; Yan et al., 2018; Ma et al., 2019; Yuan et al., 2019; Song et al., 2021). Because biphenyls have received little attention compared to PPAPs and xanthones in Clusiaceae, a comprehensive review of biphenyl chemicals in Clusiaceae is currently unavailable. Since 1974, a total of 69 new biphenyls (Tables 1–5) have been identified from Clusiaceae, all of which are structurally distinctive and possess various bioactive effects. It is obvious that a summary of the biphenyls in Clusiaceae would be beneficial for the utilization of these compounds. Therefore, we would like to sum up the development of biphenyls in the chemistry and biological activity from Clusiaceae, with the aim to provide a discussion of their structural diversity and medicinal potential.

**TABLE 1:** Biphenyls from Clusiaceae and associated cytotoxicities.

Compound		Source	Cell lines	IC_50_ (µM)^a^	Ref
1	[1,1-biphenyl]-2-(3-methyl-2-butenyl)-3-methoxy4,4,5,6-tetraol	*Garcinia bancana*			Rukachaisirikul et al. (2005a)
		*Garcinia mckeaniana*	KB	_	Auranwiwat et al. (2021)
**2**	Cylindrobiphenyl B	*Garcinia cylindrocarpa*	KB, HeLa S3, MCF-7, HepG2, HT-29	_	Sukandar et al. (2018)
**3**	Aucuparin	*Garcinia linii*	P-388	3.21^b^	Chen et al. (2004)
			HT-29	5.39^b^	Chen et al. (2004)
		*Garcinia schomburgkiana*	KB, HeLa S3, HepG2, MCF-7	_	Sukandar et al. (2016b)
		*Berberis koreana; Chaenomeles sinensis*	A549, SK-OV-3, SK-MEL-2, and HCT15	_	Kim et al. (2009); Kim et al. (2016)
		*Chaenomeles speciosa*	XF498	_	Suh et al. (2017)
**4**	Garcibiphenyl C	*Garcinia linii*			Chen et al. (2006)
		*Garcinia speciosa*	P388, KB, Col-2, BCA-1, Lu-1, ASK; Jurkat, NALM-6, K562 and HPB-ALL	_	Pailee et al. (2018); Ito et al. (2013)
**5**	Nigrolineabiphenyl A	*Garcinia* nigrolineata			Rukachaisirikul et al. (2005b)
		*Nicotiana tabacum*	NB4	6.8 ± 0.7	Zhou et al. (2015)
			A549	7.4 ± 0.8	Zhou et al. (2015)
			SHSY5Y	_	Zhou et al. (2015)
			PC-3	6.2 ± 0.6	Zhou et al. (2015)
			MCF-7	8.0 ± 0.8	Zhou et al. (2015)
		*Garcinia schomburgkiana*	Jurkat, NALM-6, K562 and HPB-ALL	_	Ito et al. (2013)
**6**	Nigrolineabiphenyl B	*Garcinia* nigrolineata			Rukachaisirikul et al. (2005b)
		*Garcinia schomburgkiana*	SW620	0.36	Mungmee et al. (2013)
			BT474, HepG2, KATO-III, CHAGO	_	Mungmee et al. (2013)
		*Nicotiana tabacum*	NB4	7.3 ± 0.7	Zhou et al. (2015)
			A549	_	Zhou et al. (2015)
			SHSY5Y	6.0 ± 0.6	Zhou et al. (2015)
			PC-3	5.7 ± 0.6	Zhou et al. (2015)
			MCF-7	6.2 ± 0.5	Zhou et al. (2015)
		*Garcinia schomburgkiana*	Jurkat, NALM-6, K562 and HPB-ALL	_	Ito et al. (2013)
**7**	Garcinuntabiphenyl A	*Garcinia nuntasaenii*	P-388, KB, HT-29, MCF-7, A549, ASK	_	Chaturonrutsamee et al. (2018)
**8**	Garcinuntabiphenyl B	*Garcinia nuntasaenii*	P-388	37.87	Chaturonrutsamee et al. (2018)
			KB	41.94	Chaturonrutsamee et al. (2018)
			HT-29, MCF-7, A549, ASK	_	Chaturonrutsamee et al. (2018)
**9**	Garcinuntabiphenyl C	*Garcinia nuntasaenii*	P-388	39.04	Chaturonrutsamee et al. (2018)
			KB, HT-29, MCF-7, A549, ASK	_	Chaturonrutsamee et al. (2018)
**10**	Schomburgbiphenyl	*Garcinia schomburgkiana*	SW620, BT474, HepG2, KATO-III, CHAGO	_	Mungmee et al. (2013)
		*Garcinia bracteata*	NB4, A549, SHSY5Y, PC-3, MCF-7	_	Li et al. (2015)
**11**	Schomburgbiphenyl B	*Garcinia schomburgkiana*	Jurkat, NALM-6, K562 and HPB-ALL	_	Ito et al. (2013)
**12**	Garciosine A	*Garcinia speciosa*	P-388, Col-2, BCA-1, Lu-1, ASK	_	Pailee et al. (2018)
		*Garcinia cylindrocarpa*	KB	89.05 ± 3.11	Sukandar et al. (2018)
			HeLa S3, MCF-7, HepG2, HT-29	_	Sukandar et al. (2018)
**13**	Garciosine B	*Garcinia speciosa*	P-388	18.48^c^	Pailee et al. (2018)
			KB, Col-2, BCA-1, Lu-1, ASK	-	Pailee et al. (2018)
**14**	Doitungbiphenyl A	*Garcinia sp. twigs*	MCF-7	102.52	Siridechakorn et al. (2014)
			KB	_	Siridechakorn et al. (2014)
**15**	Doitungbiphenyl B	*Garcinia sp. twigs*	MCF-7	_	Siridechakorn et al. (2014)
			KB	_	Siridechakorn et al. (2014)
**16**	Bractebiphenyl A	*Garcinia bracteata*	NB4, PC-3	_	Li et al. (2015)
			A549	5.8	Li et al. (2015)
			SHSY5Y	8.6	Li et al. (2015)
			MCF-7	7.3	Li et al. (2015)
**17**	Bractebiphenyl B	*Garcinia bracteata*	NB4, A549, PC-3	_	Li et al. (2015)
			SHSY5Y	7.9	Li et al. (2015)
			MCF-7	6.4	Li et al. (2015)
**18**	Bractebiphenyl C	*Garcinia bracteata*	NB4	5.8	Li et al. (2015)
			PC-3	3.6	Li et al. (2015)
			A549	8.8	Li et al. (2015)
			SHSY5Y	2.7	Li et al. (2015)
			MCF-7	8.5	Li et al. (2015)
**19**	Mckeaniabiphenyl	*Garcinia mckeaniana*	KB	_	Auranwiwat et al. (2021)
**20**	Garmultine A	*Garcinia multiflora*	HeLa	30.0^d^	Tian et al. (2017)
			MCF-7	23.5^d^	Tian et al. (2017)
			A-549	12.4^d^	Tian et al. (2017)
			MGC-803, COLO-205	33.3^d^	Tian et al. (2017)
**21**	Garmultine B	*Garcinia multiflora*	HeLa	30.8^d^	Tian et al. (2017)
			MCF-7	23.5^d^	Tian et al. (2017)
			A-549	20.5^d^	Tian et al. (2017)
			MGC-803	_	Tian et al. (2017)
			COLO-205	38.1^d^	Tian et al. (2017)
**22**	Garmultine C	*Garcinia multiflora*	HeLa	38.4^d^	Tian et al. (2017)
			MCF-7, A-549	_	Tian et al. (2017)
			MGC-803	36.7^d^	Tian et al. (2017)
			COLO-205	39.5^d^	Tian et al. (2017)
**23**	Multiflorabiphenyl B1	*Garcinia multiflora*	HeLa, SGC7901, TE1, HCT116, Capan 2	_	Fu et al. (2015)
**24**	Multiflorabiphenyl D	*Garcinia multiflora*	HeLa, SGC7901, TE1, HCT116, Capan 2	_	Fu et al. (2015)
**25**	Oblongifoliagarcinine A	*Garcinia oblongifolia*	A549, HL-60	_	Wu et al. (2008)
		*Garcinia multiflora*	HeLa, COLO-205	_	Tian et al. (2017)
			MCF-7	18.9^d^	Tian et al. (2017)
			A-549	36.6^d^	Tian et al. (2017)
			MGC-803	38.4^d^	Tian et al. (2017)
**26**	Oblongifoliagarcinine B	*Garcinia oblongifolia*	A549, HL-60	_	Wu et al. (2008)
		*Garcinia multiflora*	HeLa	30^d^	Tian et al. (2017)
			MCF-7	18.4^d^	Tian et al. (2017)
			A-549	35.1^d^	Tian et al. (2017)
			MGC-803	33.1^d^	Tian et al. (2017)
			COLO-205	35.0^d^	Tian et al. (2017)
**27**	Oblongifoliagarcinine C	*Garcinia oblongifolia*	A549, HL-60	_	Wu et al. (2008)
		*Garcinia multiflora*			Wang et al. (2018)
**28**	Oblongifoliagarcinine D	*Garcinia oblongifolia*	A549, HL-60	_	Wu et al. (2008)
**29**	3-methoxy-5-methoxycarbonyl-4-hydroxy-biphenyl	*Garcinia oligantha*	NB4	_	Liu et al. (2015)
			PC-3	_	Liu et al. (2015)
			MCF-7	4.8	Liu et al. (2015)
			A549	6.2	Liu et al. (2015)
			SHSY5Y	7.1	Liu et al. (2015)
**30**	Schomburgbiphenyl A	*Garcinia schomburgkiana*	Jurkat, NALM-6, K562 and HPB-ALL	_	Ito et al. (2013)
**31**	Clusiparalicoline A	*Clusia paralicola*	KB	3.8 ± 0.4^e^	Seo et al. (1999)
**32**	Clusiparalicoline B	*Clusia paralicola*	KB	4.5 ± 2.2^e^	Seo et al. (1999)
**33**	Clusiparalicoline C	*Clusia paralicola*	KB	5.3 ± 1.6^e^	Seo et al. (1999)
**34**	Cylindrobiphenyl A	*Garcinia cylindrocarpa*	KB, HeLa S3, MCF-7, HepG2, HT-29	_	Sukandar et al. (2018)
**35**	Garcibiphenyl A	*Garcinia linii*	P-388	10.2^b^	Chen et al. (2004)
			HT-29	13.5^b^	Chen et al. (2004)
**36**	Garcibiphenyl B	*Garcinia linii*	P-388	6.63^b^	Chen et al. (2004)
			HT-29	12.7^b^	Chen et al. (2004)
**37**	Garcibenzopyran	*Garcinia linii*	P-388	3.98^b^	Chen et al. (2004)
			HT-29	6.90^b^	Chen et al. (2004)
**38**	Multiflorabiphenyl C	*Garcinia multiflora*	HeLa, SGC7901, TE1, HCT116, Capan 2	_	Fu et al. (2015)
**39**	Garciosine C	*Garcinia speciosa*	P388	32.36^c^	Pailee et al. (2018)
			KB, Col-2, BCA-1, Lu-1, ASK, HeLa; S-3, MCF-7, HepG2, HT-29	_	Pailee et al. (2018); Sukandar et al. (2018)

^a^ IC_50_ (half-maximal inhibitory concentration), EC_50_ (concentration for 50% of maximal effect) or ED_50_ (median effective doses) with specific values in the reference is listed in the table. The dash in the table means cytotoxicity was tested with no IC_50_ value provided.

^b^ ED_50_ (in µg/mL) was recorded.

^c^ ED_50_ (in µM) was recorded.

^d^ IC_50_ (in µg/mL) was recorded.

^e^ EC_50_ (in µg/mL) was recorded.

**TABLE 2:** Biphenyls from Clusiaceae and associated antibacterial activity.

Compound		Source	Bacteria	MIC (µg/mL)^a^	Ref
**1**	[1,1-biphenyl]-2-(3-methyl-2-butenyl)-3-methoxy4,4,5,6-tetraol	*Garcinia bancana*	MRSA^b^	64	Rukachaisirikul et al. (2005a)
**3**	Aucuparin	*Garcinia linii*	*Mycobacterium tuberculosis* 90–221387	52.3 ± 6.4	Chen et al. (2006)
		*Kielmeyera coriacea*	*Bacillus subtilis*	3.12	Cortez et al. (2002)
			*S. aureus* ^c^	12.5	Cortez et al. (2002)
			*Pseudomonas aeruginosa, Escherichia coli*	_	Cortez et al. (2002)
**6**	Nigrolineabiphenyl B	*Garcinia nigrolineata*			Rukachaisirikul et al. (2005b)
		*Garcinia fusca*	*Helicobacter pylori* ATCC 43504	226.3^f^	Nontakham et al. (2014)
			*Helicobacter pylori* DMST 20165	56.5^f^	Nontakham et al. (2014)
			*Helicobacter pylori* HP40	226.3^f^	Nontakham et al. (2014)
**15**	Doitungbiphenyl B	*Garcinia sp. twigs*			Siridechakorn et al. (2014)
		*Garcinia esculenta*	MSSA^d^-Newman, MRSA-USA300 LAC and USA400 MW2, VISA^e^ Mu50	50	Zheng et al. (2021)
**40**	Garciesculenbiphenyl A	*Garcinia esculenta*	MSSA^d^-Newman, MRSA-USA300 LAC and USA400 MW2, VISA^e^ Mu50	50	Zheng et al. (2021)
**41**	Garciesculenbiphenyl B	*Garcinia esculenta*	MSSA^d^-Newman, MRSA-USA300 LAC and USA400 MW2, VISA^e^ Mu50	>100	Zheng et al. (2021)
**42**	*Garcinia*cowol	*Garcinia cowa*	MRSA-SK1	_	Siridechakorn et al. (2012)
			*S. aureus* ^c^ TISTR 1466	_	Siridechakorn et al. (2012)
			*Escherichia coli* TISTR 780	128	Siridechakorn et al. (2012)
			*Salmonella typhimurium* TISTR 292	128	Siridechakorn et al. (2012)
**43**	2,2-dimethyl-3,5-dihydroxy-7-(4-hydroxyphenyl)chromane	*Clusia burlemarxii*	*Micrococcus luteus* ATCC 10240	25	Ribeiro et al. (2011)
			*S. aureus* ^c^ ATCC 6538	50	Ribeiro et al. (2011)
			*Bacillus subtilis* ATCC 6633, *Streptococcus mutans* ATCC 5175	100	Ribeiro et al. (2011)
			*Escherichia coli* ATCC 94863*, Salmonella choleraesuis* ATCC 14028*, Pseudomonas aeruginosa*	_	Ribeiro et al. (2011)
**44**	Garcibiphenyl D	*Garcinia linii*	*Mycobacterium tuberculosis* 90-221387	50 ± 4.2	Chen et al. (2006)
**45**	Garcibiphenyl E	*Garcinia linii*	*Mycobacterium tuberculosi*s 90-221387	25.4 ± 3.1	Chen et al. (2006)
**46**	(S)-3-Hydroxygarcibenzopyran	*Garcinia linii*	*Mycobacterium tuberculosis* 90-221387	_	Chen et al. (2006)

^a^ MIC (minimum inhibitory concentration) with specific values in the reference is listed. The dash in the table means antibacterial activity was tested with no MIC value provided.

^b^ MRSA, means methicillin-resistant *Staphylococcus aureus*.

^c^ *S. aureus* means *Staphylococcus aureus*.

^d^ MSSA, means methicillin-susceptive *Staphylococcus aureus*.

^e^ VISA, means vancomycin-intermediate resistant *Staphylococcus aureus*.

^f^ MIC (in µM) was recorded.

**TABLE 3:** Biphenyls from Clusiaceae and associated anti-tobacco mosaic virus (anti-TMV) activity at the concentration of 20 μM.

Compound		Source	Inhibition rates to TMV (%)	Ref
**3**	Aucuparin	*Garcinia tetralata*	24.5 ± 2.8	Hu et al. (2016)
**5**	Nigrolineabiphenyl A	*Garcinia nigrolineata*		Rukachaisirikul et al. (2005b)
		*Garcinia tetralata*	23.5 ± 2.7	Hu et al. (2016)
**6**	Nigrolineabiphenyl B	*Garcinia nigrolineata*		Rukachaisirikul et al. (2005b)
		*Garcinia tetralata*	18.9 ± 2.9	Hu et al. (2016)
**10**	Schomburgbiphenyl	*Garcinia schomburgkiana*		Mungmee et al. (2013)
		*Garcinia bracteata*	16.7 ± 2.6	Li et al. (2015)
**14**	Doitungbiphenyl A	*Garcinia sp. twigs*		Siridechakorn et al. (2014)
		*Garcinia bracteata*	15.8 ± 2.0	Li et al. (2015)
		*Nicotiana tabacum*	18.8 ± 2.5	Shang et al. (2014)
**15**	Doitungbiphenyl B	*Garcinia sp. twigs*		Siridechakorn et al. (2014)
		*Garcinia bracteata*	21.5 ± 2.4	Li et al. (2015)
		*Nicotiana tabacum*	24.8 ± 2.6	Shang et al. (2014)
**16**	Bractebiphenyl A	*Garcinia bracteata*	15.5 ± 2.3	Li et al. (2015)
**17**	Bractebiphenyl B	*Garcinia bracteata*	18.2 ± 2.7	Li et al. (2015)
**18**	Bractebiphenyl C	*Garcinia bracteata*	28.4 ± 2.5	Li et al. (2015)
**25**	Oblongifoliagarcinine A	*Garcinia oblongifolia*		Wu et al. (2008)
		*Garcinia bracteata*	21.0 ± 2.5	Li et al. (2015)
**47**	Multiflorabiphenyl B2	*Garcinia multiflora*	28.3	Xu et al. (2016)
**48**	Tetralatabiphenyl A	*Garcinia tetralata*	21.5 ± 2.6	Hu et al. (2016)
**49**	Tetralatabiphenyl B	*Garcinia tetralata*	22.8 ± 2.8	Hu et al. (2016)
**50**	Tetralatabiphenyl C	*Garcinia tetralata*	31.1 ± 3.5	Hu et al. (2016)
**51**	Multiflorabiphenyl A2	*Garcinia multiflora*	25.4	Xu et al. (2016)

**TABLE 4:** Biphenyls from Clusiaceae and associated anti-rotavirus activity.

Compound		Source	CC_50_ (μM)^a^	EC_50_ (μM)^b^	SI or TI^c^ (CC_50_/EC_50_)	Ref
**52**	Garcilancibiphenyl A	*Garcinia lancilimba*	305.3 ± 2.0	17.3 ± 0.9	17.65	Gao et al. (2018) ^f^
**53**	Garcilancibiphenyl B	*Garcinia lancilimba*	285.3 ± 1.2	19.6 ± 1.1	14.56	Gao et al. (2018) ^f^
**54**	Garcilancibiphenyl C	*Garcinia lancilimba*	175.5 ± 1.7	32 ± 1.6	5.48	Gao et al. (2018) ^f^
**55**	Multibiphenyl A	*Garcinia multiflora*	125.72 ± 6.41^d^	11.56 ± 1.13^d^	10.93 ± 1.27	Gao et al. (2016) ^g^
**56**	Multibiphenyl B	*Garcinia multiflora*	134.65 ± 8.34^d^	10.94 ± 1.65^d^	12.35 ± 1.75	Gao et al. (2016) ^g^
**57**	Multibiphenyl C	*Garcinia multiflora*	159.83 ± 7.46^d^	12.73 ± 1.75^d^	12.58 ± 1.68	Gao et al. (2016) ^g^
**58**	2-isopropenyl-6-methoxy-7-hydroxy-(4-hydroxyphenyl)-dihydrobenzofuran	*Garcinia tetralata*	164.2	12.9	12.73^e^	Ji et al. (2017) ^h^
**59**	[1.1′-biphenyl]-3-methoxy-4.4′,5-triol	*Garcinia tetralata*	185.5	12.6	14.72^e^	Ji et al. (2017) ^h^

^a^ CC_50_ means 50% value of cytotoxic concentration on MA104 cells.

^b^ EC_50_ means 50% value of effective concentration on rotavirus infected MA104 cells.

^c^ TI, means the therapeutic index.

^d^ CC_50_ or EC_50_ (in μg/mL) was recorded.

^e^ SI (Selective index) was recorded.

^f^ Using ribavirin as positive control (CC_50_ = 263.2 ± 1.9 μM, EC_50_ = 13.3 ± 0.7 μM, TI = 19.8).

^g^ Using ribavirin as positive control (CC_50_ = 274.27 ± 11.07 μM, EC_50_ = 13.61 ± 1.04 μM, TI = 20.14 ± 1.16).

^h^ Using ribavirin as positive control (CC_50_ = 263.2 μM, EC_50_ = 13.3 μM, SI = 19.8).

**TABLE 5:** Biphenyls from Clusiaceae and associated anti-syncytium activity.

Compound		Source	IC_50_ (μM)^a^	EC_50_ (μM)^b^	SI (IC_50_/EC_50_)	Ref
**4**	Garcibiphenyl C	*Garcinia linii*	—	—	—	Chen et al. (2006)
		*Garcinia speciosa*	75.57	40.17	1.88	Pailee et al. (2018) ^c^
**7**	Garcinuntabiphenyl A	*Garcinia nuntasaenii*	>507.59	301.84	>1.68	Chaturonrutsamee et al. (2018) ^d^
**8**	Garcinuntabiphenyl B	*Garcinia nuntasaenii*	390.90	131.97	2.98	Chaturonrutsamee et al. (2018) ^d^
**9**	Garcinuntabiphenyl C	*Garcinia nuntasaenii*	292.41	84.10	3.48	Chaturonrutsamee et al. (2018) ^d^
**12**	Garciosine A	*Garcinia speciosa*	107.45	56.28	1.91	Pailee et al. (2018) ^c^
**13**	Garciosine B	*Garcinia speciosa*	67.82	<14.22	>4.76	Pailee et al. (2018) ^c^
**39**	Garciosine C	*Garcinia speciosa*	151.97	20.23	7.51	Pailee et al. (2018) ^c^

^a^ IC_50_ = dose of compound that inhibited 50% metabolic activity of uninfected 1A2 cells.

^b^ EC_50_ = dose of compound that reduced by 50% syncytium formation by ^ΔTat/Rev^MC99 virus in 1A2 cells.

^c^ Using azidothymidine as positive control (IC_50_ > 10^–2^ μM, EC_50_ = 3.95 × 10^–3^ μM, SI > 2.53).

^d^ Using ribavirin as positive control (IC_50_ = 3.74 × 10^–8^ μM, EC_50_ > 1.37 × 10^–8^ μM, SI > 2.73).

## 2 Diverse biphenyls from Clusiaceae and their bioactivity

Tables 1–5 outlines the biphenyls isolated from Clusiaceae and their bioactivities. As shown in Table 1, almost two-thirds of the compounds were examined for cytotoxicity, however, only a few of them were revealed with moderate bioactivities. Nine compounds (**3**, **5–6**, **16–18**, **29, 31** and **37**) showed relatively good potency against the cells tested, with IC_50_ values below 10 μM. Tables 2, 3 list the substances that were tested for antibacterial and anti-tobacco mosaic virus (anti-TMV) activity. Compounds **3**, **6**, and **45** performed well in the anti-microbial test, with MIC values of roughly 20 μg/mL against corresponding bacteria. In terms of the capacity to inhibit tobacco mosaic virus, two-thirds of the compounds examined exhibited good activity, with inhibition rates greater than 20%, while the positive control ningnanmycin generally showed inhibition rates in the range of 30%–35% (Shang et al., 2014; Hu et al., 2016). Among them, compounds **18**, **47** and **50** showed better inhibition rates, which were close to 30%. Tables 4, 5 present the results of a small number of compounds that were tested for anti-rotavirus and anti-HIV efficacy. It is obvious that all the substances under study have some potential when their therapeutic indices (TIs) or the selective indices (SIs) are compared to the corresponding positive controls. The TI value of the anti-rotavirus positive control Ribavirin is about 20 (Gao et al., 2016), and the SI value of the anti-syncytium positive control azidothymidine is larger than 2.73 (Chaturonrutsamee et al., 2018).

In the following section, the origins of the compounds, their structures, and an extensive description of their biological activities are presented based on the Tables. The isolated compounds are organized based on the structures and their bioactivities.

### 2.1 Biphenyls with associated cytotoxicities

**FIGURE 3:**
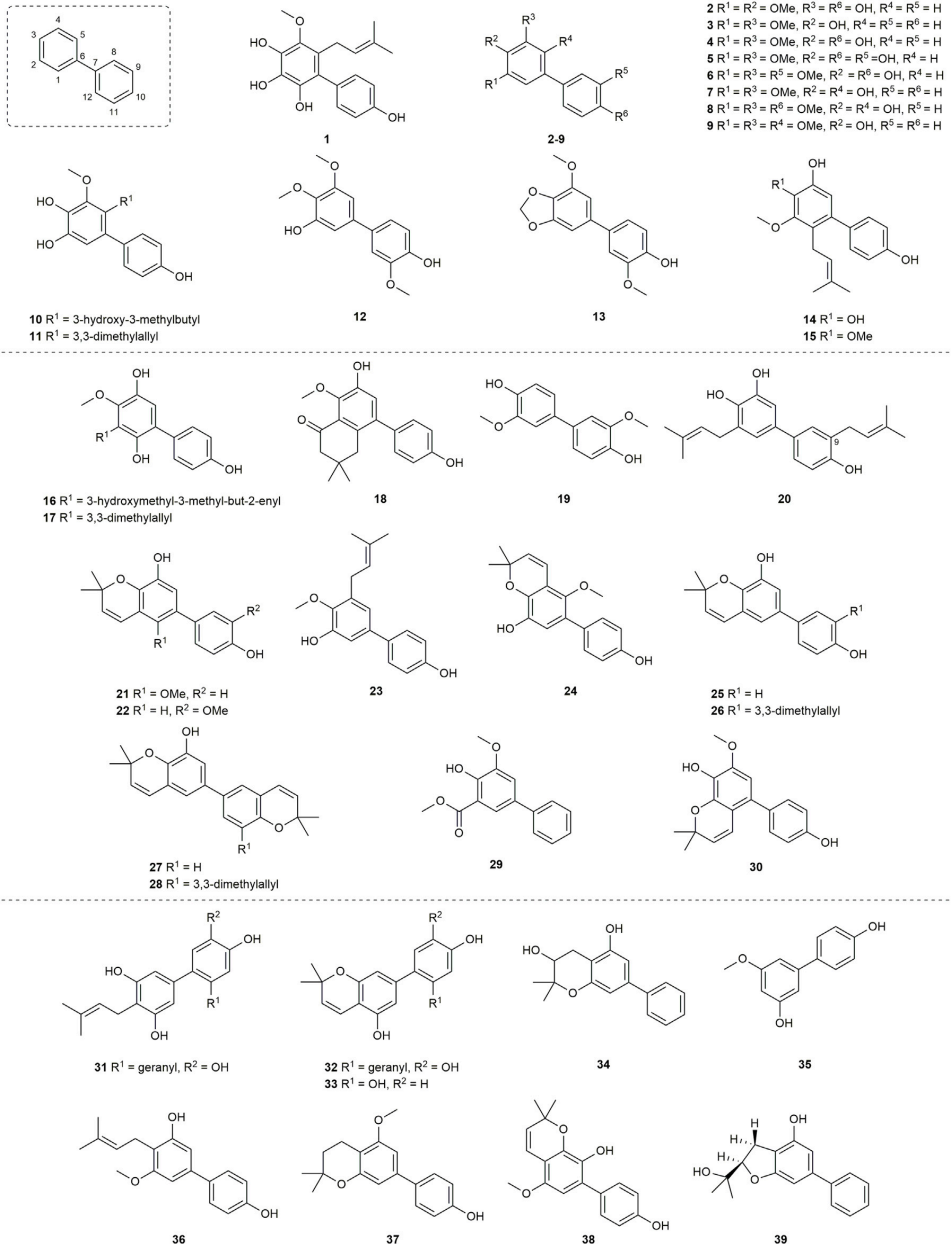
Structures of biphenyls **1–39**.

A total of 39 compounds (Figure 3) were isolated and assayed for cytotoxicity from Clusiaceae. Three of them were from *Clusia paralicola* G. Mariz and the rest of the compounds were from *Garcinia* genus. The cytotoxicity of the substances is detailed below by categorizing them into three groups based on the number of hydroxyl groups and substitution sites on the structures.

#### 2.1.1 Biphenyls with 2,3,4-trihydroxyl group

The biphenyl structures in this section contain three hydroxyl groups at the C2, C3, and C4 positions, and their cytotoxicities have been evaluated on several cancer cell lines.

A biphenyl was isolated from the methanol extract of twigs and leaves of *Garcinia bancana* MIQ. from Southern Thailand, which was determined as [1.1′-biphenyl]-2-(3-methyl-2-butenyl)-3-methoxy-4.4′,5,6-tetraol (**1**) (Figure 3) (Rukachaisirikul et al., 2005a). The source of biphenyl **1**, *Garcinia bancana,* is a member of the Clusiaceae family that is native to Southeast Asian countries including Thailand, Malaysia and Indonesia (Whitmore, 1973; Rukachaisirikul et al., 2005a). The *n*-hexane extract of the *Garcinia bancana* leaves showed antioxidant activity according to 1,1-diphenyl-2-picrylhydrazyl (DPPH) and ferric reducing antioxidant power (FRAP) tests (Putri et al., 2018). In the research of Auranwiwat and co-workers, cytotoxicity against KB cells of **1** was tested, but **1** was inactive to both cells with IC_50_ > 50 µM (Table 1) (Auranwiwat et al., 2021).

*Garcinia cylindrocarpa* Kosterm is native to Indonesia’s Maluku Island, where it is known as “Kogbirat” and is used as a fever remedy in traditional Indonesian medicine (Sukandar et al., 2016a). Tip-pyang and others explored *Garcinia cylindrocarpa* for sustained research on bioactive compounds of *Garcinia* and attained cylindrobiphenyl B (**2**) (Figure 3). By utilizing the 3-(4,5-dimethylthiazol-2-yl)-2,5-diphenyl-2,3-dihydro-1H-tetrazol-3-ium bromide (MTT) colorimetric technique, biphenyl **2** showed no activity against KB, HeLa S3, MCF-7, HepG2, or HT-29 cancer cell lines. (Table 1) (Sukandar et al., 2018).

Aucuparin (**3**) (Figure 3), a constitutive component from the heartwood of *Sorbus aucuparia* (Erdtman et al., 1961), also could be extracted and separated from the *Garcinia* species (Chen et al., 2004; Mungmee et al., 2013; Hu et al., 2016). Although the structure of **3** is very simple, many studies have explored its biological properties such as antifungal (Cortez et al., 1998; Song et al., 2021), anti-TMV (Hu et al., 2016), anti-inflammatory, as it is a natural phytoalexin (Kokubun et al., 1995). Given that **3** has attracted a lot of attention and has been studied by many groups, some of its biological features including cytotoxicity, antibacterial activity and anti-inflammatory were summarized individually in the following sections.

A lot of groups have probed its cytotoxic effects against diverse cell lines, and **3** showed different levels of cytotoxicity against P-388 (IC_50_ = 3.21 μg/mL (Chen et al., 2004)), HT-29 (IC_50_ = 5.39 μg/mL (Chen et al., 2004)), XF498, A549, SK-OV-3, SK-MEL-2, HCT15 (IC_50_ > 30 µM (Kim et al., 2009; Kim et al., 2016; Suh et al., 2017)), KB, HeLa S3, HepG2 and MCF-7 (IC_50_ > 100 µM (Sukandar et al., 2016b)) cells (Table 1).

**SCHEME 1:**
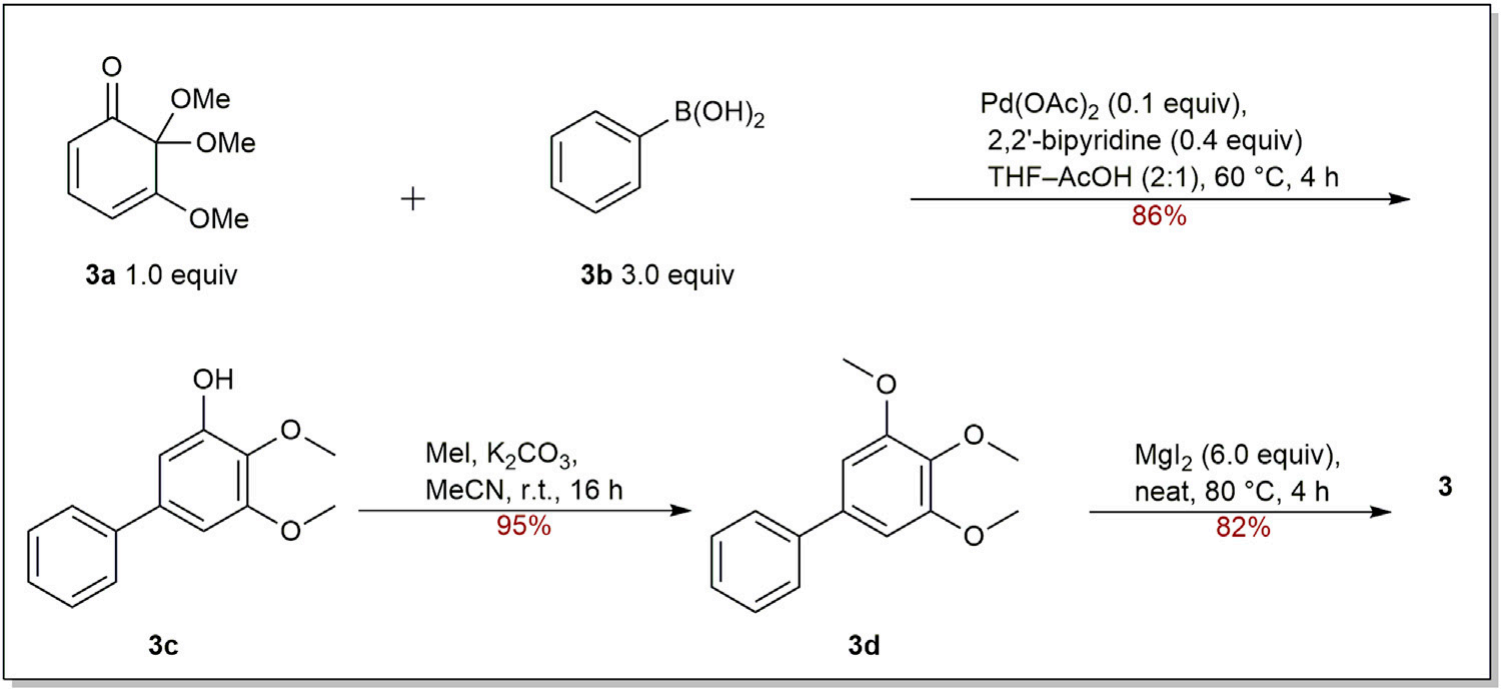
Synthesis of biphenyl **3**

For the synthesis of oxygenated biphenyls, the tandem Michael addition reaction followed by aromatization reaction was developed by Chittimalla group. They successfully applied this methodology in the synthesis of **3** (Scheme 1) (Chittimalla et al., 2015). At the beginning, cyclohexa-2,4-dienone (**3a**) and phenylboronic acid (**3b**) were used as the starting materials to make **3c**, which underwent quantitative methylation by reacting with MeI and K_2_CO_3_ in acetonitrile to give trimethoxybiphenyl **3d**. After selective demethylation, biphenyl **3** was accomplished in 82% yield.

**SCHEME 2:**
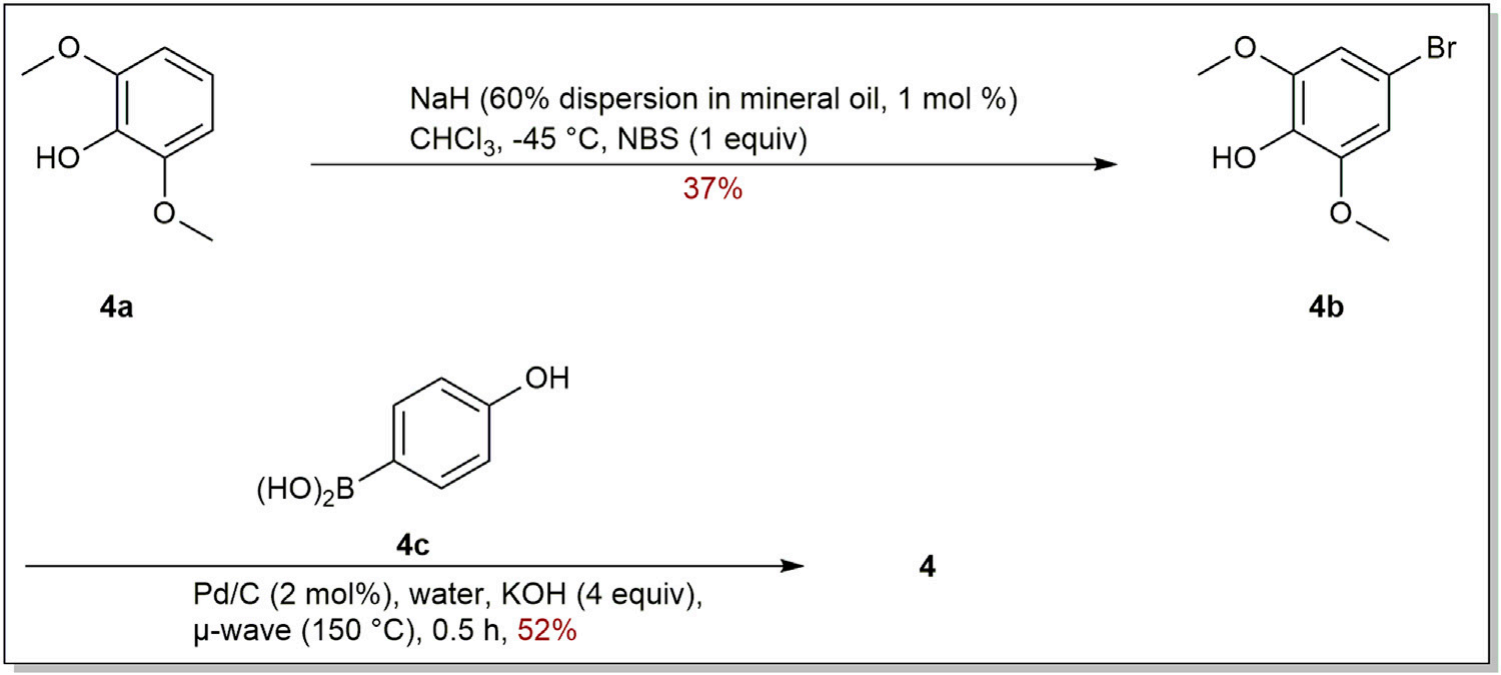
Synthesis of biphenyl **4**

*Garcinia linii* is an endemic evergreen tree that sprang up in Langyu land along with the Green island of Taiwan and contains xanthones, biphenyls, and other compounds. Chen et al. (2019). Chen group made significant contributions to the separation of biphenyls from the root of *Garcinia linii*. They analyzed and identified garcibiphenyl C (**4**) (Figure 3), by way of spectral analyses in 2006 (Chen et al., 2006). Biphenyl **4** was tested for its cytotoxic activity against P388, KB, Col-2, BCA-1, Lu-1, ASK, NALM-6, but it was found to be ineffective against these cells (Table 1) (Ito et al., 2013; Pailee et al., 2018). With respect to the synthesis of **4**, the strategy reported by Schmidt group was featured with protecting-group free strategy. (Scheme 2). At the outset, bromoarene (**4b**) was produced from 2,6-dimethoxyphenol (**4a**) *via* the bromination of **4a** (Lee et al., 2004). Then **4b** was reacted with (4-hydroxyphenyl) boronic acid (**4c**) in the presence of Pd/C catalyst in water to give **4** in 52% yield. In addition, this research reported that it could get access to diverse biphenyls *via* Suzuki−Miyaura cross coupling (Schmidt and Riemer, 2014).

*Garcinia nigrolineata* Planch. Ex T. Anderson, known as kandis in the local, is widely distributed in Malaysia (Mohd Norfaizal et al., 2014). Many xanthones have been isolated (Rukachaisirikul et al., 2003a; Rukachaisirikul et al., 2003c) from this plant. On the other hand, only two biphenyls, nigrolineabiphenyls A-B (**5–6**) (Figure 3) were obtained from *Garcinia nigrolineata* by Rukachaisirikul in 2005 (Rukachaisirikul et al., 2005b). The cytotoxicities of **5-6** on a series of tumor cell lines were studied including SW620, BT474, HepG2, CHAGO, NB4, A549, NALM-6 and so on (Table 1) (Ito et al., 2013; Mungmee et al., 2013; Zhou et al., 2015). Biphenyls **5-6** showed weak to moderate inhibitory activity towards most of the cell lines mentioned above, while **6** displayed stronger cytotoxicity against SW620 cells with the IC_50_ values of 0.36 µM (Mungmee et al., 2013).

The first investigation to identify the phytochemical of *Garcinia nuntasaenii* Ngerns. and Suddee attained the isolation of garcinuntabiphenyls A-C (**7–9**) (Figure 3), which were evaluated for cytotoxicity (Chaturonrutsamee et al., 2018). *Garcinia nuntasaenii* is distributed in northeastern Thailand and commonly named “Chang-nga-ek”. It is a dioecious shrub growing with white flowers and green fruits. Its roots could be used for relieving muscle pain in Thai folk medicine (Ngernsaengsaruay and Suddee, 2016). The cytotoxic effects of biphenyls (**7–9**) against a panel of cultured mammalian cancer cell lines, including P-388, KB, HT-29, MCF-7, A549, ASK, were generally weak. Only the cytotoxicity of **8** to P-388 (IC_50_ = 37.87 µM) and KB (IC_50_ = 41.94 µM), as well as **9** to KB (IC_50_ = 39.04 µM) were detected and quantified (Table 1).

A new biphenyl, schomburgbiphenyl (**10**) (Figure 3), was obtained as a white solid from the wood of *Garcinia schomburgkiana* Pierre and determined as [1.1′-biphenyl]-2-(3-hydroxy-3-methylbutyl)-3-methoxy-4.4′,5-triol (Mungmee et al., 2013). Its source, *Garcinia schomburgkiana,* is a medium-sized tree widely scattered in Vietnam, Laos, Cambodia, and Thailand and called Ma-dan in Thailand. It has previously been reported to possess the activity of DNA polymerase Inhibitory, antioxidant and anti-diabetic properties (Le et al., 2016; Gonsap and Vongsak, 2019; Thummajitsakul et al., 2020). Biphenyl **10** was analyzed for its cytotoxic activity against five human cancer cell lines, but it was not active in the test (Table 1) (Mungmee et al., 2013). Another study carried out by Hu also tested its cytotoxicities using the other five kinds of tumor cell lines (NB4, A549, SHSY5Y, PC-3, MCF-7) in 2015. And it could be known that all IC_50_ values were above 10 µM from this research (Table 1) (Li et al., 2015).

Investigating the EtOAc extract of the stems of *Garcinia schomburgkiana* led to the fractionation and the elucidation of a new biphenyl derivative, schomburgbiphenyl B (**11**) (Figure 3), as colorless oil. The compound (**11**) was screened for the ability to repress the growth of several leukemia cell lines containing Jurkat, NALM-6, K562 and HPB-ALL. It was found that biphenyl **11** showed limited cytotoxicity against NALM-6 cells, with cell viability ranging from 73% to 80% at the concentration of 50 µM (Table 1) (Ito et al., 2013).

Two biphenyls, garciosines A-B (**12–13**) (Figure 3), were isolated, established, and confirmed from *Garcinia speciose* Wall by employing extensive spectroscopic methods and single crystal X-ray diffraction analysis (Pailee et al., 2018). *Garcinia speciose*, namely “PhaWaa” in Thailand, is an indigenous plant that can be used for the therapy of blood disorders, skin wounds, inflammation, laxative and so on (Sakunpak and Panichayupakaranant, 2012; Sangsuwon and Jiratchariyakul, 2015). Garciosines A-B (**12–13**) were examined with cytotoxic properties against a panel of cancer cell lines (P388, KB, Col-2, BCA-1, Lu-1, ASK, HeLa, S-3, MCF-7, HepG2, HT-29). The activity of **12–13** to restrain these tumor cells was not prominent. The IC_50_ value of **12** against KB cells was 89.05 ± 3.11 µM, and the IC_50_ value of **13** against P388 cells was 18.48 µM (Table 1) (Pailee et al., 2018; Sukandar et al., 2018).

The separation of doitungbiphenyls A (**14**) and B (**15**) (Figure 3) from the acetone extract of the twigs of *Garcinia* sp. was achieved for the first time by Laphookhieo and co-works. By virtue of 1D and 2D NMR spectroscopy as well as HR-EI-MS, they were elucidated to contain prenylated chains in the skeleton. Obviously, **15** is formed from the methylation of **14**. Their bioactivities to suppress the reproduction of the tumor cells were tested by using KB and MCF-7 cell lines. However, the two compounds were inactive against the two cell lines, and **15** displayed weak inhibition against the MCF-7 cell line with an IC_50_ value of 102.52 μM (Table 1) (Siridechakorn et al., 2014).

#### 2.1.2 Biphenyls with 2,3-dihydroxyl group

The structural similarity of the compounds described in this section is characterized by the presence of two adjacent hydroxyl substituents at the C2, C3 positions. Their cytotoxicities against diverse tumor cell lines were summarized in the following.

Mixed forests of *Garcinia bracteata* C. Y. Wu ex Y. H. Li are commonly distributed on limestone hills of Yunnan and Guangxi Province of China (Li et al., 2011). To search for new bioactive metabolites, Hu et al. examined the chemical components of the twigs from *Garcinia bracteata*. Consequently, three biphenyls, namely bractebiphenyls A-C (**16–18**) (Figure 3), were extracted and separated by using 70% aqueous acetone. Their structures were elucidated by spectroscopic methods, such as UV spectrum, IR spectrum and 1D and 2D NMR techniques. As for the experimental data of cytotoxic abilities, three compounds displayed moderate cytotoxicities against five cell lines (NB4, A549, SHSY5Y, PC-3, and MCF-7), with IC_50_ values typically below 10 µM. Among these compounds, biphenyl **18** was the most potential with high cytotoxicities against A549 and PC-3 cells (IC_50_ = 3.6 and 2.7 µM, respectively) (Table 1) (Li et al., 2015).

For the aim of searching for undiscovered natural products of *Garcinia mckeaniana* Craib, the constituents belonging to the flower and twig extracts of this plant were investigated by Auranwiwat and others and a biphenyl (**19**) was found (Figure 3) in 2021 (Auranwiwat et al., 2021). *Garcinia mckeaniana*, also called Xen mu, is a common plant that is widespread in the tropical secondary forests of Sapa town and Son La province of Vietnam in particular. So far, a few studies have been conducted on the phytochemistry and biological properties of *Garcinia mckeaniana* (Auranwiwat et al., 2016; Ha et al., 2021; Nguyen et al., 2021; Thi Thu et al., 2021). The biphenyl (**19**) was denominated as mckeaninabiphenyl and had been synthesized previously (Sato and Mikawa, 1960). This was the first time that it was reported as a natural phytochemical. Its structure is symmetrically featured as 4.4′-dihydroxy-2.2′-dimethoxybiphenyl, which is like biphenyls that were isolated from fungi formerly (Li et al., 2017; Zhu et al., 2019). Its cytotoxicity was tested, but it was inactive against KB cells with IC_50_ value > 50 µM in assays (Table 1).

Yuan group systematically discovered and structurally characterized Garmultines A-C (**20–22**) (Figure 3) from *Garcinia multiflora* Champion ex Bentham (Tian et al., 2017), which contains PPAPs with apoptosis-inducing or cytotoxic properties (Chien et al., 2008; Liu et al., 2010; Fan et al., 2015; Tian et al., 2016; Yang et al., 2020) and others (Wang et al., 2018). Compounds **21** and **22** are proved to be isomers. The cytotoxic capacities of the three novel natural biphenyls (**20–22**) were evaluated (Tian et al., 2017). Their cytotoxicities were tested on five human tumor cell lines (HeLa, MCF-7, A-549, MGC-803, and COLO-205), which turned out that these biphenyls (**20–22**) were moderate against the five cells with the IC_50_ values ranging from 12.4 to > 40 μg/mL (Table 1). It concluded that an additional prenyl chain at C9 (**20**) could make compound **20** more active in inhibiting tumor cells.

Two biphenyls, multiflorabiphenyls B1 (**23**) and multiflorabiphenyl D (**24**) (Figure 3) were obtained from the acetone-extracted leaves of *Garcinia multifora* through bioassay-directed fractionation. The cytotoxic abilities of compounds **23–24** against five human cancer cell lines (HeLa, SGC7901, TE1, HCT116, and Capan 2) were evaluated with IC_50_ values > 10 µM (Table 1) (Fu et al., 2015).

*Garcinia oblongifolia* Champ. ex Benth. is a medium-sized shrub mainly distributed in tropical areas of southern China and northern Vietnam and has been used to reduce the pains of burns and diminish inflammation (Liu et al., 2016). Xanthones, PPAPs and other bioactive components have been found in this plant (Hamed et al., 2006; Zhang et al., 2016). Four new biphenyls, oblongifoliagarcinines A–D (**25–28**) (Figure 3), were acquired from samples of *Garcinia oblongifolia* collected in Guangxi province of China and structurally determined on the basis of spectroscopic analysis. The cytotoxic effects of **25–28** were assessed against the tumor cell lines A549 and HL-60 (Table 1). It turned out that biphenyls **25–28** were inactive toward these two cancer cells *in vitro* (Wu et al., 2008). Whereas, compounds **25** and **26** showed weak influence on the growth of HeLa, MCF-7, A-549, MGC-803, and COLO-205 cells with IC_50_ values ranging from 18.4 to > 40 μg/mL (Tian et al., 2017).

The new biphenyl (**29**) (Figure 3) was obtained and confirmed as 3-methoxy-5-methoxycarbonyl-4-hydroxy-biphenyl after the extraction, separation and purification from the stems of *Garcinia oligantha* Merrill (Liu et al., 2015). *Garcinia oligantha* is a tall shrub mainly growing in the Guangdong and Hainan provinces of China and northern Vietnam (Li et al., 2011). The plant was usually explored for the bioactive xanthones possessing diverse activity such as cytotoxic property and suppressing convulsant behavior (Tang et al., 2016; Tang et al., 2019; Gong et al., 2020; Yang et al., 2021). The biphenyl (**29**) was measured for its cytotoxic effects against NB4, A549, SHSY5Y, PC-3 and MCF-7 tumor cells. The results showed **29** could modestly exert the repression toward tumor proliferation with IC_50_ values of 7.1, 6.2 and 4.8 µM against SHSY5Y, A549 and MCF-7 cells, respectively (Table 1) (Liu et al., 2015).

Schomburgbiphenyl A (**30**) (Figure 3) was also extracted as colorless oil from the stems of *Garcinia schomburgkiana* at the same time with Schomburgbiphenyl B (**11**). Like compound **11**, **30** was accessed for the cytotoxicity against a series of leukemia cell lines containing Jurkat, NALM-6, K562 and HPB-ALL cells. It (**30**) was not very effective to repress the proliferation of these tumor cells in study (Table 1) (Ito et al., 2013).

#### 2.1.3 Biphenyls with 2, 4-dihydroxyl group

Biphenyls with bis-hydroxyl substituted at the C2 and C4 positions and their cytotoxic testing results are outlined in this section.

Three novel biphenyls clusiparalicolines A-C (**31–33**) (Figure 3) were obtained by the bioassay-guided fractionation from the roots of *Clusia paralicola* G. Mariz (Seo et al., 1999), which is a native species distributed in Brazil (POWO, 2022). These three biphenyls were tested for cytotoxicity against KB cells. The results showed that biphenyls **31–33** could inhibit the proliferation of KB cells modestly (Table 1). The report also assessed the DNA strand scission ability to evaluate their antineoplastic potential. Compounds **31** and **32** demonstrated considerable DNA strand scission action. The DNA relaxation rates were 77% and 65%, respectively, at the concentration of 2.5 g/mL (the rate of bleomycin at 0.025 g/mL was about 50%). Compound **33** was observed to be inactive. Looking into their structures, the ring generated by cyclization involving the prenyl and hydroxyl groups distinguishes compounds **31** and **32**. And compound **33** lacks the catechol and geranyl moiety in comparison to compounds **31** and **32**. As a result, it is possible that the 3,3-dimethylallyl group and the hydroxyl group of compound **31** have no effect on DNA strand scission activity, because there was no significant difference in DNA strand scission activity between compounds **31** and **32**. Furthermore, based on previous work by Wall and Wani’s group (Huang et al., 1996), it is confirmed that the presence of the catechol and geranyl moiety in **31** and **32** may be connected with DNA strand-scission activity.

**SCHEME 3:**
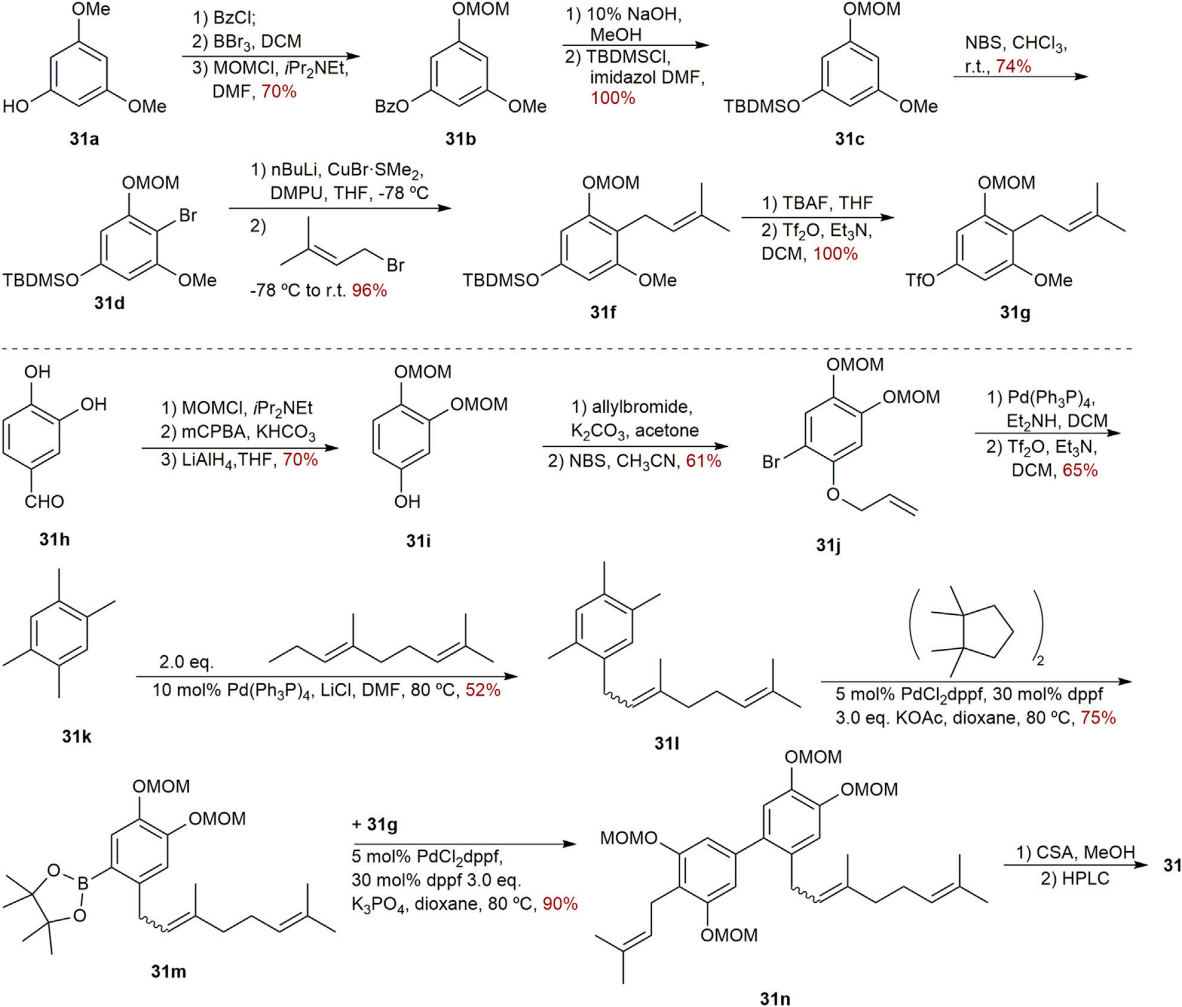
Synthesis of biphenyl **31**.

The first synthesis of biphenyl **31** was successfully finished in 2002 by Fukuyama’s team (Scheme 3). The design of two geranylated and prenylated phenols is needed for the synthesis of clusiparalicoline A (**31**) since it is not entirely symmetrical. Beginning with *O*-dimethylphloroglusinol **31a**, the preparation of left part was undertaken. After the protection, bromination, *C*-alkylation, removal of the TBDMS and subsequent triflation, the left part of **31g** with a triflate group was provided quantitatively. Starting with the commercially available 3,4-dihydroxybenzaldehyde **31h**, the protection of the catechol group, Baeyer–Villiger oxidation of the aldehyde, and reduction were performed to afford compound **31i**. Then, the generated phenolic group was protected as an allyl ether, and subsequent bromination, deallylation, palladium-catalyzed Stille reaction yielded compound **31l**. Next, it was successful to convert the bromide **31l** into a pinacol boronic ester **31m** using the Suzuki-Miyaura protocol. Following that, Suzuki coupling between **31g** and **31m** was carried out smoothly, yielding the coupling product **31n** in 90% yield. At last, following the deprotection of all MOM groups and separation by HPLC, the desired biphenyl **31** was synthesized (Takaoka et al., 2002).

Cylindrobiphenyl A (**34**) was found by Tip-pyang and others during the investigation of exploring *Garcinia cylindrocarpa* (Figure 3). Its (**34**) cytotoxicity against KB, HeLa S3, MCF-7, Hep G2, or HT-29 cancer cell lines was assayed by MTT *in vitro*. But it was inactive to all tested cells (Table 1) (Sukandar et al., 2018).

Garcibiphenyls A-B (**35–36**) (Figure 3) were separated and determined from *Garcinia linii* by Chen group in 2004 (Chen et al., 2004). Compounds **35** and **36** were assessed to figure out their cytotoxicities against two tumor cells. Their ED_50_ value against P-388 were 10.2 μg/mL, 6.63 μg/mL, respectively. And the ED_50_ values of **35–36** against HT-29 were 13.5 μg/mL, 12.7 μg/mL, respectively (Table 1) (Chen et al., 2004).

Garcibenzopyran (**37**) (Figure 3) was found and identified with **35–36** from *Garcinia linii* during the same investigation conducted by Chen group (Chen et al., 2004). The ED_50_ values against P-388 and HT-29 cell lines of **37** were 3.98 μg/mL and 6.90 μg/mL, respectively, which supported that **37** could be slightly beneficial for anti-cancer and anti-proliferation therapy (Table 1) (Chen et al., 2004).

Multiflorabiphenyl C (**38**) (Figure 3), an isomer of multiflorabiphenyl D (**24**), was obtained from the leaves of *Garcinia multifora*. The cytotoxic activity of compound **38** against five human cancer cell lines (HeLa, SGC7901, TE1, HCT116, and Capan 2) was evaluated, and the results showed that its IC_50_ values were all above 10 µM (Table 1) (Fu et al., 2015).

Garciosine C (**39**) (Figure 3) was another biphenyl isolated and established from *Garcinia speciose* by extensive spectroscopic methods in 2018 (Pailee et al., 2018). The cytotoxic ability of garciosine C (**39**) was examined on a panel of cancer cell lines (P388, KB, Col-2, BCA-1, Lu-1, ASK, HeLa, S-3, MCF-7, HepG2, HT-29). In fact, biphenyl **39** cannot inhibit tumor cells effectively. The IC_50_ value of **39** against P388 cell was 32.36 µM. In addition, it was inactive against the other cell lines (Table 1) (Pailee et al., 2018; Sukandar et al., 2018).

#### 2.1.4 Structure-activity relationship of biphenyls with associated cytotoxicity

A brief analysis of the structure-activity relationship for the cytotoxic activity of these functionalized biphenyls was discussed in this section based on the reference data that is currently available. In addition to the analyses mentioned above, the following discussion could be useful for the research of the biphenyls according to Table 1 and Figure 3.

Compounds **5** and **6** (Figure 3) were both derived from the *Garcinia* nigrolineata (Rukachaisirikul et al., 2005b). And it could be known from the research of Hu group and Suttisri group that compound **6** had a higher inhibitory effect on tested PC-3, MCF-7 and SW620 cells than that of compound **5** (Table 1) (Mungmee et al., 2013; Zhou et al., 2015). It could be inferred that the methoxyl substitution on R^5^ (C9) had a better influence on the bioactivity compared to the hydroxyl substitution. From the structures and their cytotoxicity of compounds **7** and **8**, **7** and **9** (Figure 3 and Table 1), it indicated that methoxylation on the R^6^ (C10) or the R^4^ (C5) positions may improve the cytotoxic effect. Although the cytotoxic effects of compounds **14** and **15** were not greatly outstanding, it suggested that compound **14** was slightly superior to compound **15** (Table 1) (Siridechakorn et al., 2014). Thus, it is obvious that a *meta*-hydroxyl substitution at the 3,3-dimethylallyl chain in their structures may be more advantageous for activity. The comparison of biphenyls **16** and **17** suggested that hydroxylation of the terminal carbon of the 3,3-dimethylallyl group did not have much effect on their activity (Li et al., 2015). Similarly, the experimental data for compounds **25** and **26** showed that the 3,3-dimethylallyl substitution at the C9 position did not significantly influence activity (Tian et al., 2017).

### 2.2 Biphenyls with associated antibacterial activity

**FIGURE 4:**
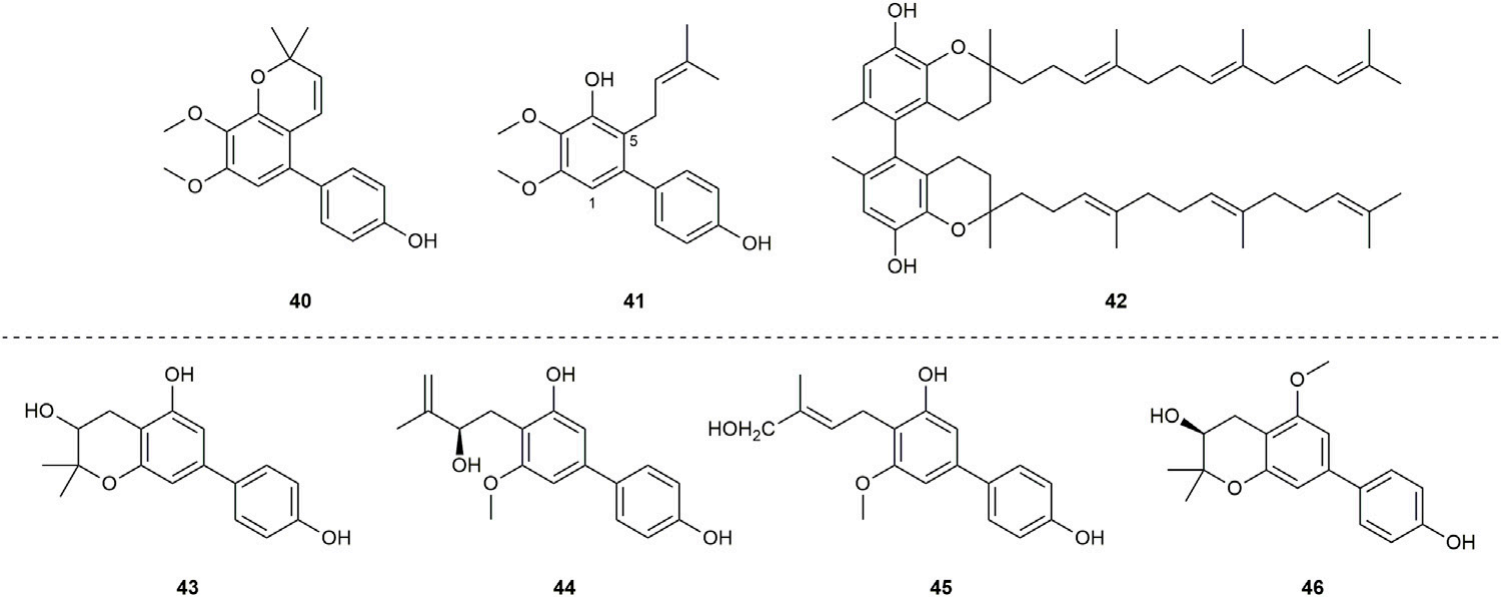
Structures of biphenyls **40–46**.

A total of 11 biphenyls (Figure 3 and Figure 4) isolated from Clusiaceae were evaluated for antibacterial activity, four of which (**1**, **3**, **6**, **15**) were listed in the cytotoxicity section above. Except for compound **43** (Figure 4), which was produced from *Clusia burlemarxii* Bittrich, another 10 compounds were isolated and extracted from *Garcinia*. They were also described below according to their structures.

#### 2.2.1 Biphenyls with 2,3,4-trihydroxyl or 2,3,5-trihydroxyl group

In addition to the cytotoxicity, several 2,3,4-trihydroxy-substituted biphenyls were also subjected to antimicrobial evaluation. The antimicrobial activity of these biphenyls and another undescribed 2,3,5-trihydroxybiphenyl are summarized in this section.

The biphenyl (**1**) (Figure 3) exhibited weak antibacterial activity to Methicillin-resistant *Staphylococcus aureus* (MRSA) with MIC values of 64 μg/mL (Rukachaisirikul et al., 2005a) (Table 2). In terms of antibacterial activity (Table 2), a study indicated that biphenyl **3** (Figure 3) could suppress Gram-positive bacteria, which showed antimicrobial activity against *Bacillus subtilis* with MIC values of 3.12 μg/mL and against *S. aureus* with a MIC value of 12.5 μg/mL, respectively. It has no effect on Gram-negative bacteria including *Pseudomonas aeruginosa* and *Escherichia coli* (MIC ≥100 μg/ml). Meanwhile, penicillin-resistant *S. aureus* (PRSA) strains (MIC = 6.25 μg/mL) seemed to be more sensitive to **3** than penicillin-susceptible *S. aureus* (PSSA) strains (MIC = 12.5 μg/mL), which was advantageous for clinical drug development. The observation that anti-bactericidal activity of **3** functionalized in a dose-dependent manner was proved by investigating the kinetics of bactericidal activity against *S. aureus* at six concentrations of **3** (0.25 ×, 0.5 ×, 1 ×, 2 ×, 4 × and 8 × the MIC). It was surprising that there was no regrowth of bacteria after monitoring for up to 24 h (Cortez et al., 2002). The suppressive degree of **3** against *Mycobacterium tuberculosis* 90 ± 221387 was also tested to evaluate its antitubercular activity and it turned out that **3** played a weak role in antitubercular effect (Chen et al., 2006). With respect to biphenyl **6** (Figure 3), a study tested the antibacterial activity of **6** against three different strains of *Helicobacter pylori* (*H. pylori*) (Table 2). The results indicated **6** could restrain *H. pylori* in a gentle manner with MIC values of 226.3 µM, 56.5 µM, 226.3 µM against *H. pylori* ATCC 43504, *H. pylori* DMST 20165 and *H. pylori* HP40, respectively (Nontakham et al., 2014). On the other hand, compound **15** (Figure 3) was indicated to show anti-staphylococcal activity with MIC values at 50 μg/mL towards series of *S. aureus* strains (Table 2) (Zheng et al., 2021).

Two previously unknown biphenyls, garciesculenbiphenyls A-B (**40–41**) (Figure 4) were recently isolated and identified from *Garcinia esculenta* Y. H. Li, an endemic tree mainly distributed in Yunnan province of China (Zhu et al., 2014). Compounds **40** and **41** are two chemicals that are structurally related. Compound **41** could be converted to **40** after oxo-cyclization. These two compounds were tested for antibacterial activity against MSSA-Newman, two MRSA strains USA300 LAC and USA400 MW2, and VISA-Mu50 (Table 2). As it revealed, compound **40** presented weak anti-staphylococcal activity with MIC values at 50 μg/mL, whereas compound **41** did not show obvious anti-staphylococcal activity (Zheng et al., 2021).

#### 2.2.2 Biphenyls with 2, 3-dihydroxyl group

The compound **42** (Figure 4) was isolated from *Garcinia cowa* Roxb. *Garcinia cowa,* also known as Cha muang in Thailand. It is a plant that can produce a variety of bioactive substances and is used as an antipyretic folk medicine (na Pattalung et al., 1994; Likhitwitayawuid et al., 1998; Mahabusarakam et al., 2005; Panthong et al., 2006) (+)-Garciniacowol (**42**), a dimeric dihydrobenzopyran with highly symmetrical structure, was found and identified from the stembarks of *Garcinia cowa* (Siridechakorn et al., 2012). The antibacterial property of **42** was analyzed (Table 2) and the results showed that **42** was inert against Gram-positive bacteria such as *S. aureus-*TISTR 1466 and MRSA-SK1. On the other hand, **42** had a mild effect on Gram-negative bacteria such as *Escherichia coli* TISTR 780 and *Salmonella typhimurium* TISTR 292 with MIC values at 128 g/mL for both.

#### 2.2.3 Biphenyls with 2, 4-dihydroxyl group

2,2-dimethyl-3,5-dihydroxy-7-(4-hydroxyphenyl)chromane (**43**) (Figure 4) was extracted and determined from the trunk of *Clusia burlemarxii* Bittrich, a shrub distributed in Brazil. The research carried out the preliminary tests *in vitro* to determine the inhibitory effect of biphenyl **43** against a series of bacteria. The results of the initial analysis revealed that biphenyl **43** significantly inhibited all tested Gram-positive bacteria which was stronger against *Micrococcus luteus* (MIC = 25 μg/mL^−1^) and *S. aureus* (MIC = 50 μg/mL^−1^). However, it was weaker against *Bacillus subtilis* and *Streptococcus mutans* with MIC values at 100 μg/mL^−1^. Additionally, it had no effect on Gram-negative bacteria (Table 2) (Ribeiro et al., 2011).

Garcibiphenyls D-E (**44–45**) (Figure 4) were discovered at the same time as garcibiphenyl C (**4**) from *Garcinia linii* by Chen group in 2006 (Chen et al., 2006). Regarding to biphenyls **44** and **45**, their anti-tubercular effects *in vitro* were evaluated against *Mycobacterium tuberculosis* 90 ± 221387. Both of them showed moderate activity with MIC values at 50.3 ± 4.2 μg/mL, 25.4 ± 3.1 μg/mL, respectively (Table 2) (Chen et al., 2006).

(*S*)-3-hydroxygarcibenzopyran (**46**) (Figure 4) was found and identified simultaneously with biphenyls **4**, **44–45** from *Garcinia linii* (Chen et al., 2006). It is apparent that **46** is the derivative of **37** with an additional hydroxyl on the pyran ring. Although the antibacterial effect of biphenyl **46** was investigated, the testing result displayed that biphenyl **46** could not inhibit *Mycobacterium tuberculosis* 90 ± 221387 effectively (MIC >100 μg/mL) (Table 2) (Chen et al., 2006).

#### 2.2.4 Structure-activity relationship of biphenyls with antibacterial activity

According to the study of Xu group in 2021, it could be known that biphenyls **15** (Figure 3), **40** (Figure 4) had comparable antibacterial capacities. Furthermore, both biphenyl **15** and **40** were more active than compound **41** (Figure 4) (Zheng et al., 2021). Hence, it could assume that the antibacterial activity may be stronger when the 3,3-dimethylallyl chain was positioned at *para*-position of the hydroxyl group (C1) or cyclized with the hydroxyl group rather than at the *ortho*-position of hydroxyl group (C5). Conclusions could also be obtained by comparing the activity of compounds **44** and **45**, **44** and **46** (Figure 4; Table 2): 1) 3-hydroxymethyl-3-methyl-but-2-enyl substitution might be more beneficial for antibacterial activity than 2-hydroxy-3-methyl-but-3-enyl substitution; 2) the cyclization of 2-hydroxy-3-methyl-but-3-enyl may impair the antibacterial abilities of the compounds.

### 2.3 Biphenyls with associated anti-TMV activity

**FIGURE 5:**
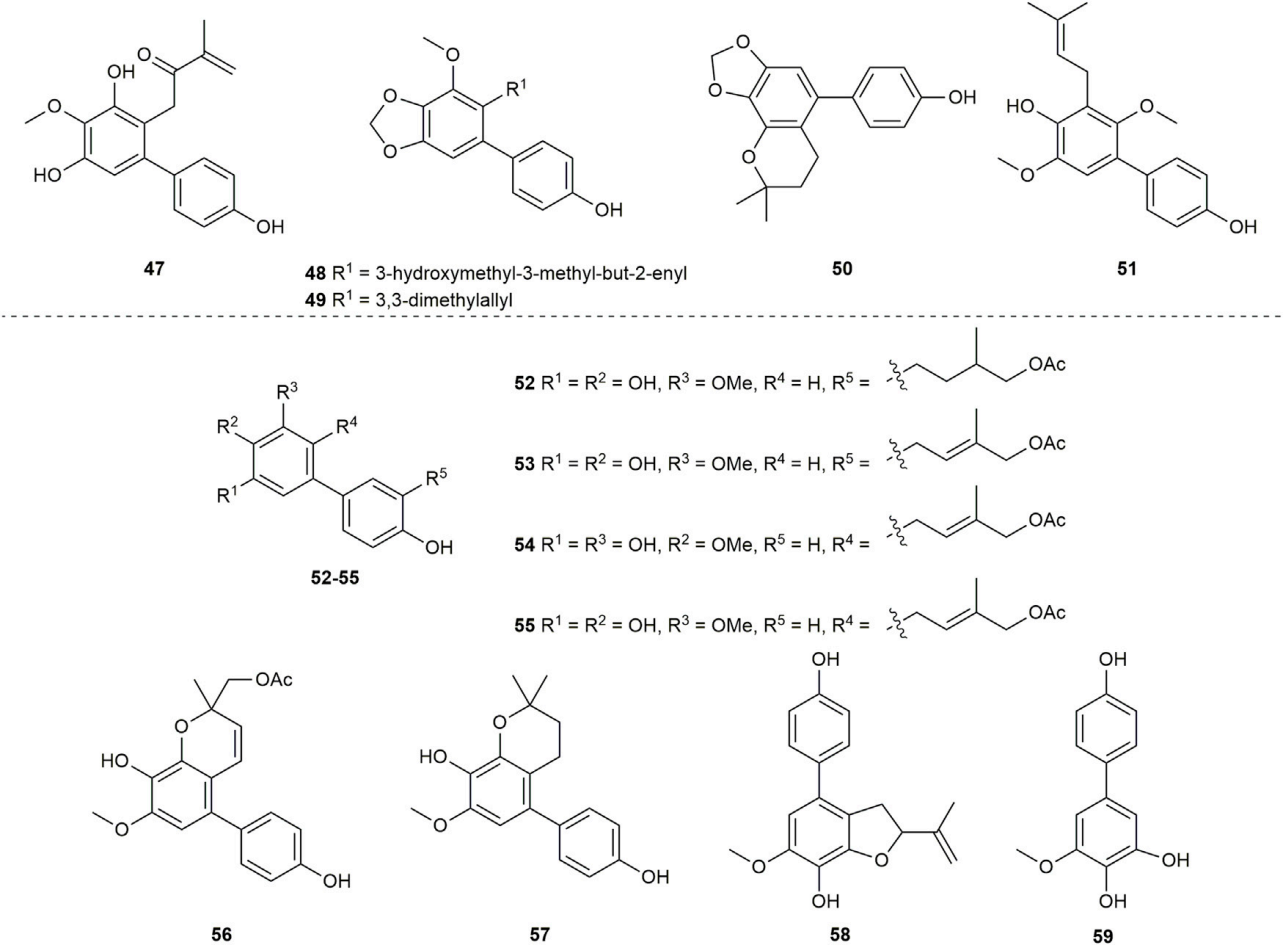
Structures of biphenyls **47–59**.

The ability of 15 different substances (Figure 3 and Figure 5) to inhibit the tobacco mosaic virus was examined. And *Garcinia* genus was the source of all compounds. They can be separated into the following two major groups based on their structural features.

#### 2.3.1 Biphenyls with 2,3,4-trihydroxyl group

Despite with a simple structure, biphenyl **3** (Figure 3) has a wide range of activities. It could moderately suppress the tobacco mosaic virus with the inhibition rates of 24.5 ± 2.8% (Table 3) (Hu et al., 2016). Additionally, biphenyl **5** and **6** (Figure 3) derived from compound **3** presented modest anti-TMV properties. Their inhibition rates were 23.5 ± 2.7% and 18.9 ± 2.9% at the concentration of 20 μM, respectively (Table 3) (Hu et al., 2016). The anti-TMV ability of **10** (Figure 3) was also assessed at the concentration of 20 μM, which showed weak inhibitory capacity that its inhibition rate was 16.7 ± 2.6% (Table 3) (Li et al., 2015). With respect to the anti-TMV activity of biphenyls (**14–15**) (Figure 3), they were found to be modest against the tobacco mosaic virus with the inhibition rates ranging from 15.8 ± 2.0% to 24.8 ± 2.6% (Table 3) (Shang et al., 2014; Li et al., 2015).

One new biphenyl, multiflorabiphenyl B2 (**47**) (Figure 5), found in the twigs of *Garcinia multiflora* was analyzed and elucidated *via* spectral techniques. Assays were managed to evaluate its anti-TMV activity, which demonstrated that **47** was potential in anti-TMV with inhibition rates of 28.3%, which was similar to the inhibition rates of the positive control, ningnanmycin (a commercial product for plant disease in China, 33.5%) (Table 3) (Xu et al., 2016).

Three biphenyl derivatives, tetralatabiphenyls A–C (**48–50**) (Figure 5), were isolated and elucidated structurally for the first time from the ethanol extract of the twigs of *Garcinia tetralata* C. Y. Wu ex Y. H. Li. *Garcinia tetralata* is a plant peculiarly distributed in the south and southwest Yunnan province in China. Up to now, the phytochemical investigation of this plant was not paid much attention (Wang et al., 2008; Guo et al., 2011). To evaluate the bioactive characterizations of biphenyls **48–50**, the activity to resist the tobacco mosaic virus was assayed. Tetralatabiphenyl C (**50**) showed the strongest activity among these biphenyls (**48–50**) with an inhibition rate of 31.1 ± 3.5% at the concentration of 20 μM, while the inhibition rates of **48** and **49** were 21.5 ± 2.6%, 22.8 ± 2.8% respectively (Table 3) (Hu et al., 2016).

#### 2.3.2 Biphenyls with 2,3-dihydroxyl group

The anti-TMV inhibition rates of **16–18** (Figure 3) at the concentration of 20 µM were 15.5 ± 2.3%, 18.2 ± 2.7%, 28.4 ± 2.5% (Table 3), respectively, while **18** showed similar inhibition rates compared with ningnanmycin (30.2%). In addition, this study also tested the anti-TMV activity of biphenyl **25** (Figure 3) and it showed that **25** could inhibit tobacco mosaic virus moderately with the inhibition rate of 21.0 ± 2.5% at the concentration of 20 µM (Table 3) (Li et al., 2015). Multiflorabiphenyl A2 (**51**) (Figure 5) was another biphenyl isolated from the twigs of *Garcinia multiflora*. Like biphenyl **47** (Figure 5), it was subject to the tests for evaluating its anti-TMV activity. The results exhibited that it was a mild inhibitor for tobacco mosaic virus with inhibition rates of 25.4% (Table 3) (Xu et al., 2016).

#### 2.3.3 Structure-activity relationship of biphenyls with anti-TMV activity

Contrary to the cytotoxicity, compound **5** had higher anti-tobacco mosaic virus activity than compound **6** (Figure 3; Table 3). It could be considered that the hydroxyl group at the R^5^ (C9) position may be helpful to increase the anti-TMV activity. Since compound **3** and compound **5** exhibited similar activity, it presumed that the adjacent double hydroxyl group at C9 and C10 had no impact on the compounds’ potential to inhibit the tobacco mosaic virus. The results of the anti-TMV activity tests for compounds **14** and **15** were also in conflict with the results of the cytotoxic activity tests. Since biphenyl **15** was shown to have a higher rate of inhibition than biphenyl **14** in both studies (Shang et al., 2014; Li et al., 2015), it can be deduced that methoxylation of the hydroxyl group at the *meta*-isopentenyl position in the structures was advantageous for enhancing the resistance to tobacco mosaic virus (Figure 3; Table 3). However, the tobacco mosaic virus inhibition rates of compounds **16** and **17** were also similar, in agreement with their cytotoxic test results. This suggested that the shift from 3,3-dimethylallyl group to 3-hydroxymethyl-3-methyl-but-2-enyl chain in their structures does not affect their activity levels (Figure 3; Table 3).

### 2.4 Biphenyls with associated anti-rotavirus activity

The biphenyls in the Clusiaceae family that have anti-rotavirus effect are all from the genus *Garcinia*. They all feature a basic structural backbone of 2,3,4-trihydroxyl substitution.

*Garcinia lancilimba* C. Y. Wu ex Y. H. Li is a small tree mainly distributed in humid dense forests of southern Yunnan province. A number of prenylated xanthones and biphenyls were isolated from this plant (Yang et al., 2007; Han et al., 2008; Gao et al., 2018). Garcilancibiphenyls A-C (**52–54**) (Figure 5), which are structurally characterized by the acetoxyprenyl group on a benzene ring, were identified *via* spectroscopic evidence from the extract of the air-dried and powdered stems of *Garcinia lancilimba* at room temperature. In the report, the anti-rotavirus activity (Table 4) of **52–54** was tested on MA104 cells in terms of CC_50_, EC_50_ and the TI (CC_50_/EC_50_). It was evident that **52** and **53** were potential for medicinal development against rotavirus with TI values above 10 (17.65, 14.56, 19.8 for **52**, **53**, Ribavirin, respectively) (Gao et al., 2018).

Multibiphenyls A-C (**55–57**) were three new biphenyls isolated from *Garcinia multiflora* Champion ex Bentham (Figure 5) (Gao et al., 2016). The distinct structures of these biphenyls are interestingly correlated. Apparently, **57** could be obtained from the deacetylation of **56**, while **56** might be the product derived from the cyclization of **55**. The potential of **55–57** to prevent the cytopathic effects caused by rotavirus was quantified in MA104 cells for the sake of assessing their bioactive significance and competence. The determination of antiviral activity was displayed as the values of CC_50_, EC_50_ and TI, together with ribavirin serving as a positive control. The results showed that all TI values were greater than 10, demonstrating the good anti-rotavirus potential of these biphenyls (**55–57**) (Table 4).

Two new biphenyls (**58–59**) (Figure 5) were yielded in a continuous search for more chemical components from *Garcinia tetralata*. Biphenyl **58** was determined as 2-isopropenyl-6-methoxy-7-hydroxy-(4-hydroxyphenyl)-dihydrobenzofuran, which can be known as the first biphenyl substituted with 2-isopropenyl dihydrobenzofuran. Another one (**59**) was recorded as [1.1′-biphenyl]-3-methoxy-4.4′,5-triol following the extensive analysis of spectroscopy techniques. The compounds (**58–59**) were screened to figure out their anti-rotavirus activity in MA104 cells. Both of them exhibited good anti-rotavirus potential with SI values were above 10 (Table 4) (Ji et al., 2017).

Except for compound **54**, the other compounds tested for anti-rotavirus activity had TI or SI values greater than 10 (Table 4). Comparing the structures of biphenyls **54** and **55,** it can be inferred that a double hydroxyl substitution next to the prenylated chain would favour the safety and efficacy of the compounds against rotavirus. While compound **55** can be converted to compound **56** *via* chain cyclization, this change has no influence on the activity (Figure 5).

### 2.5 Biphenyls with associated anti-HIV activity

A total of 7 biphenyls (Figure 3) were tested for their anti-HIV ability. They have been all mentioned above. Among them, six compounds had 2,3,4-trihydroxyl substituted structures.

#### 2.5.1 Biphenyls with 2,3,4-trihydroxyl group

Probing the anti-HIV ability of **4** (Figure 3) was practiced by methods of syncytium inhibition assay and reverse transcriptase (RT) assay. Biphenyl **4** exhibited moderate activity in the syncytium inhibition assay. It could reduce 50% of syncytium formation at the concentration of 40.17 µM and its SI (IC_50_/EC_50_) was 1.88 in the model of 1A2 cells infected by ^ΔTat/Rev^MC99 virus (Table 5). However, its repressive effect on HIV-1 reverse transcriptase was not very good. Its inhibition rate was only 8.57% at the concentration of 200 μg/mL. Therefore, its IC_50_ was not determined in the anti-HIV-1 RT assay (Pailee et al., 2018).

As for the anti-HIV activity of the biphenyls (**7–9**) (Figure 3), they presented various efficacy in the anti-HIV-1 RT assay and the syncytium inhibition assay with the system of ^ΔTat/Rev^MC99 virus and 1A2 cell line. For anti-HIV-1 RT assay of **7-9**, both **7** and **8** were mild to repress the HIV-1 reverse transcriptase with the inhibition rates of 39.54% and 49.36% at the concentration of 200 μg/mL respectively. On the other hand, **9** was very active with the inhibition rate reaching up to 91.15% at the concentration of 200 μg/mL and its IC_50_ value was 202.50 µM by contrast. In the respect of syncytium inhibition assay, all the three compounds were active to suppress the syncytium formation by ^ΔTat/Rev^MC99 virus in 1A2 cells, and similar SI scores were obtained at 1.68, 2.98, 3.48 for **7**, **8**, **9**, respectively (Table 5).

The remaining two compounds with 2,3,4-trihydroxyl substitution that were measured for anti-HIV activity were biphenyls **12** and **13** (Figure 3). With respect to their (**12–13**) anti-HIV effects, the tests were also performed by utilizing syncytium inhibition assay and the RT assay. They all demonstrated activity in the syncytium inhibition assay proceeding with the ^ΔTat/Rev^MC99 virus and 1A2 cell line system, with SI values all above one and were 1.91, >4.76 respectively (Table 5). Biphenyl **13** displayed moderate activity, whilst biphenyls **12** was inactive in the HIV-1 RT assay on the contrary. The prescreened inhibition rates of **12–13** at the concentration of 200 μg/mL were −2.53% and 53.83% respectively, but their IC_50_ values were not determined (Pailee et al., 2018).

#### 2.5.2 Biphenyls with 2,4-dihydroxyl group

Biphenyl **39** (Figure 3) was isolated and analyzed in the same study as compounds **12** and **13**. Along with biphenyls **12** and **13**, it was also subjected to both the syncytium inhibition assay and the HIV-1 RT assay. In fact, the result of the syncytium inhibition test for compound **39** was better than compound **12**. Its (**39**) SI value was above one and was 7.51 in the anti-syncytium test (Table 5). However, it (**39**) was inactive in the HIV-1 RT assay as the inhibition rate at the concentration of 200 μg/ml was 7.84%. The IC_50_ value was not determined. (Pailee et al., 2018).

#### 2.5.3 Structure-activity relationship of biphenyls with anti-HIV activity

It was clear from the aforementioned and Table 5 that the anti-HIV effects of the investigated biphenyls were generally similar. Compound **9** (Figure 3) demonstrated the best performance in the anti-HIV-1 RT assay. It was suspected that, in comparison to compound **7** (Figure 3), the methylation of hydroxyl at the R^4^(C5) site of biphenyl **9** favored to enhance inhibition ability.

### 2.6 Biphenyls with other bioactive abilities

#### 2.6.1 Biphenyls with antioxidant activity

Through 2.2′-azinobis-(3-ethylbenzthiazoline-6-sulphonate) (ABTS) radical cation decolorization assay, the anti-oxidant property of biphenyls **20–22** (Figure 3) and biphenyls **25–26** (Figure 3) was evaluated. They all turned out to have good or moderate anti-oxidant possibilities, with IC_50_ values of 7.78 µM, 38.3 µM, 8.78 µM, 7.84 µM and 29.57 µM for **20–22**, **25–26**, respectively. Ascorbic acid was employed as a positive control, with an IC_50_ value of 7.70 µM. The compound **22** appears to be stronger than **21** in terms of anti-oxidant effect, which indicated the influence of the position of the methoxy group on the phenyl. Meanwhile, although the cytotoxicities of **25** and **26** were similar, it can be seen that the bioactive properties along with the anti-oxidant ability of **26** were certainly better than **25**, which might be owing to the additional prenylated group on the phenyl of **26**. (Tian et al., 2017).

#### 2.6.2 Biphenyls with anti-inflammatory activity

Superoxide anion radical (O_2_^−^) is a predecessor of other reactive oxygen species (ROS) and could be generated by activated neutrophils in response to stimuli (Reichl et al., 2001; Selloum et al., 2001). The over-production of O_2_^−^ is a major cause for the development of inflammation and the scavenging of excess O_2_^−^ is helpful for anti-inflammatory (Kroes et al., 1992; Salvemini et al., 2001). Experiments were performed to evaluate the efficacy of **3** (Figure 3) to suppress O_2_^−^ generation induced by chemotactic peptide *N*-Formyl-L-methionyl (Met)-L-leucyl (Leu)-l-phenylalanine (Phe) (fMLP). The results shown that **3** presented effective inhibitory activity (IC_50_ = 17.0 ± 2.8 μM) on O_2_^−^ generation compared with positive control Ibuprofen (IC_50_ = 27.5 ± 3.5 μM) (Chen et al., 2009). Furthermore, the anti-neuroinflammatory activity and neuroprotective effect of **3** were probed through measuring nitric oxide (NO) inhibition in lipopolysaccharide (LPS)-stimulated murine microglia BV2 cell and determining the secretion of nerve growth factor (NGF) in C6 glial cells, respectively (Kim et al., 2016; Suh et al., 2017). It indicated that the inhibitory activity of **3** to reduce the production of NO was potent with IC_50_ values of 20.04 μM (IC_50_ values of positive control L-NMMA = 21.82 μM), and the potency of **3** in offering more NGF for neuroprotection was moderate with NGF secretion levels of 125.76 ± 4.52% (the values of positive control 6-Shogaol = 158.18 ± 6.56%). In 2021, Kim and Yoon group presented a plausible mechanism of how **3** inhibited idiopathic pulmonary fibrosis (IPF), a lung disease generating lung scarring, in a bleomycin (BLM)-induced lung fibrosis mouse model. The research found out that **3** could depress the genes of inflammation and decrease macrophage activation marker genes, along with raising the expression level of anti-fibrotic markers *in vivo*. More significantly, it revealed that **3** could hamper the production of inflammatory cytokine-induced by transforming growth factor-*β* (TGF-*β*) from macrophages and the collagen synthesis from fibroblasts. These results demonstrated that **3** could play a protective role in lung fibrosis *via* its anti-inflammatory ability and it might be a promising therapy for IPF (Lee et al., 2021). Meanwhile, biphenyl **15** (Figure 3) could slightly exert anti-inflammatory activity against cyclooxygenase-2 (COX-2), an enzyme increased may due to inflammatory events, with the IC_50_ value of 108.54 ± 0.42 μM (IC_50_ values of positive control celecoxib = 18.08 ± 0.12 μM) (Ma et al., 2019).

#### 2.6.3 Biphenyls with antimalarial activity, anti-diabetic activity, neurotrophic activity, or anti-virulence activity

Except for cytotoxicity and antibacterial, biphenyl **1** (Figure 3) showed weak antimalarial activity against Plasmodium falciparum with the IC_50_ values of 39.4 µM against TM4/8.2, and 43.1 µM against K1CB1, respectively (Auranwiwat et al., 2021).

Furthermore, the anti-diabetic activity of biphenyl **4** (Figure 3) was assessed by testing the inhibition against α-glucosidase in parallel with the inducibility of glucose consumption by 3T3-L1 cells. It could be known that the α-glucosidase inhibitory activity of **4** was not determined. However, it can induce glucose consumption without causing toxic effects with IC_50_ values of 34.7 µM. Metformin was used as positive control with an IC_50_ value of 45.4 µM (Phukhatmuen et al., 2020).

A study examined the impact of biphenyl **31** (Figure 3) on neurite outgrowth in rat cortical neurons in a primary culture. It discovered that 1 μM of biphenyl **31** could stimulate neurite outgrowth. At this concentration, its activity could be comparable to the basic fibroblast growth factor (bFGF, 40 ng/mL). However, when 10 μM of **31** was administered to neural cells, all neurons were killed during 6 days culture period. The morphology and viability of cortical neurons at a lower dosage of 0.1 μM were not affected by **31**. It was suggested that biphenyl **31** with a biaryl moiety containing alkenyl groups, would have some beneficial effects on the growth and survival of neurons (Takaoka et al., 2002).

**FIGURE 6:**
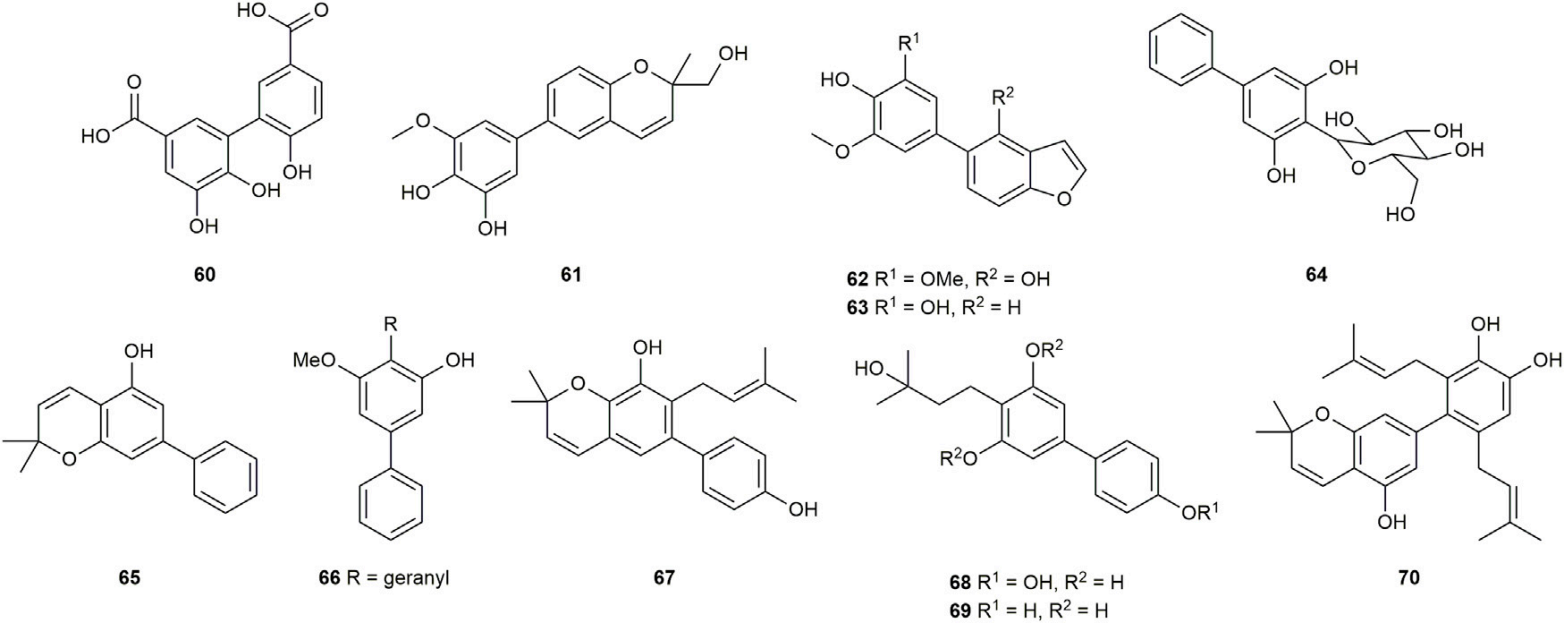
Structures of biphenyls **60–70**.

5,6,6′-trihydroxy-[1.1′-biphenyl]-3.3’dicarboxylic acid (**60**) (Figure 6) is a new natural product from the ethyl acetate extract of *Mesua ferrea* L. flower. The purpose of the research was to identify chemical inhibitors for the protein secretion system of *Salmonella enterica*, which could be thought to be responsible for the pathogenicity of the bacteria. However, biphenyl **60** lacked sufficient activity to exert inhibitory effects (Zhang et al., 2019).

### 2.7 Biphenyls with biological activity not investigated

#### 2.7.1 Biphenyls with 2,3,4-trihydroxyl group

*Garcinia kola* Heckel, a plant that is native throughout West and West Central Africa, has been intensively studied for many years due to its edible seeds, usually known as bitter kola or male kola, and medicinal potential in the treatment of a variety of infectious disorders (Hussain et al., 1982; Iwu et al., 2002; Emmanuel et al., 2022). In 1994, Niwa group isolated and identified a 6-aryl-1,2-benzopyran derivative named garcipyran (**61**) from *Garcinia Kola*. (Figure 6) (Niwa et al., 1994a).

**SCHEME 4:**
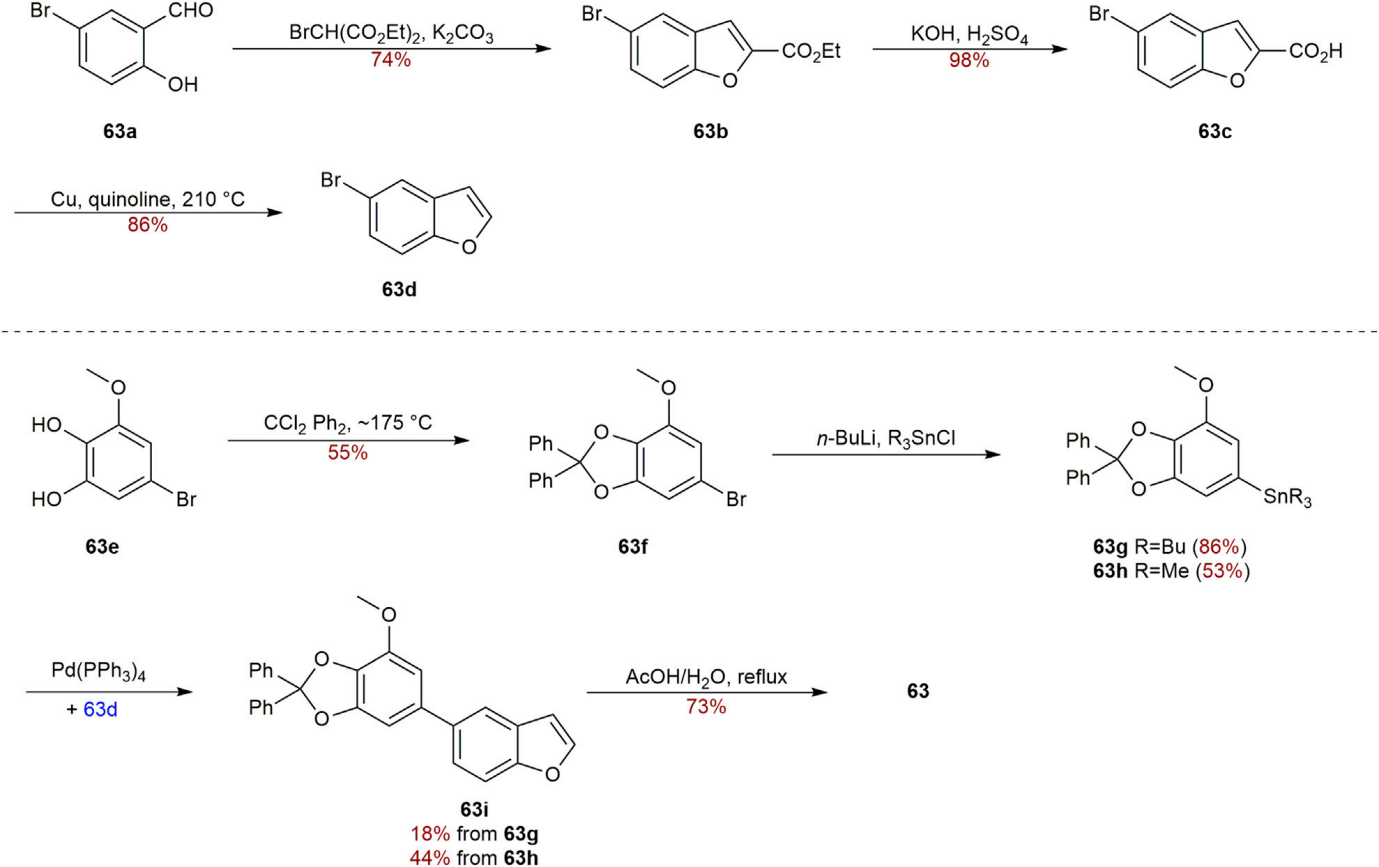
Synthesis of biphenyl **63**.

Two distinct biphenyls, in terms of garcifurans A-B (**62–63**) (Figure 6), from the methanol extract of *Garcinia Kola* roots collected in Nigeria were obtained by Niwa in 1994 (Niwa et al., 1994b). **62** and **63** were found to be the natural biphenyls claimed to possess a 5-arylbenzofuran nucleus for the first time. In order to validate the structure assignment of **63**, the total synthesis of **63** was accomplished by Kelly group in 1997 (Scheme 4). The benzofuran **63d** was prepared through condensation between 5-bromo-2-hydroxybenzaldehyde (**63a**) and bromo-malonic ester, followed by hydrolysis, and subsequent decarboxylation according to the literature (Kurdukar and Subba Rao, 1963). Next, arylstannanes **63g-63h** were gained by lithiation and quenching with Bu_3_SnCl or Me_3_SnCl after the protection of **63e** with CCl_2_Ph_2_. Afterward, the palladium-catalyzed coupling reaction between **63 g/h** and **63d** smoothly led to the precursors **63i** in 18% yield and 44% yield respectively. Heating **63i** under reflux in AcOH/H_2_O to deprotect the protecting group achieved **63** in 73% yield (Kelly et al., 1997).

#### 2.7.2 Biphenyls with 2,4-dihydroxyl group

Calophymembranside A (**64**) (Figure 6), a novel biphenyl *C*-glycoside, was identified *via* chemical analysis of the traditional Chinese medicine *Calophyllum membranaceum* Gardn. et Champ. The structure of **64** has been determined to be 3,5-dihydroxybiphenyl-4-C-*β*-glucopyranoside. It is the only biphenyl *C*-glycoside described in this article (Zou et al., 2005). Furthermore, in 2006, chromatographic treatment of the dichloromethane extract of *Clusia melchiorii* Gleason trunk resulted in the discovery of a novel biphenyl, 2,2-dimethyl-5-hydroxi-7-phenylchromene (**65**) (Figure 6) (Teixeira et al., 2006). To the best of our knowledge, *Garcinia* mangostana L (mangosteen) is a tropical tree used as medicine in Southeast Asia over the past decades and has been well-known because of its fruit, which contains a great deal of polyphenolic xanthones exhibiting various biological effects *in vitro* (Obolskiy et al., 2009). The novel geranylated biphenyl derivative 3-hydroxy-4-geranyl-5-methoxybiphenyl (**66**) (Figure 6) was separated and elucidated from extracts of root bark, stem bark and the latex collected from the green fruits of *Garcinia* mangostana by Dharmaratne and co-workers (Dharmaratne et al., 2005). Meanwhile, multiflorabiphenyl A1 (**67**) (Figure 6) was found in the stem barks of *Garcinia multiflora* in 2013 (Jing et al., 2013). And 3.4′,5-trihydroxy-4-(3-hydroxy-3-methylbutyl) biphenyl (**68**) (Figure 6) and 3,5-dihydroxy-4-(3-hydroxy-3-methylbutyl) biphenyl (**69**) (Figure 6) were extracted and analyzed from *Pentaphafangium Sofomonse* Warb (Cotterill et al., 1974).

#### 2.7.3 Others

After the separation of clusiaparalycolines A-C (**30–31**) (Figure 3), clusiaparalycoline D (**70**) (Figure 6) was isolated and elucidated during a re-examination of *Clusia paralycola* roots in 2002 (Delle Monache et al., 2002).

## 3 Conclusion and remarks

This review summarized the research progress of the biphenyls from Clusiaceae*.* The chemical structure and biological activity of 70 biphenyls were discussed in this review. Among these biphenyls, 52 compounds are found to present prenylated segment or mutative structures. Since the discovery of biphenyl **68–69** in *Pentaphafangium Sofomonse*, a total of 24 other *Garcinia* plants have been identified as sources of various biphenyls, in which *Garcinia multiflora* and *Garcinia linii* are the top two plants with the most biphenyls ascertained. The discovery of compounds **68–69** can be traced back to 1974 (Niwa et al., 1994a). And compound **3**, which was obtained from other species (Erdtman et al., 1961), could be extracted and generated from different plants of *Garcinia*.

Biphenyls **1–70** have been subjected to extensive bioactive studies, primarily involving cytotoxicity, antibacterial and antivirus activity. Compound **6**, a derivative of chemical **3** with one additional hydroxyl and methoxyl substitution, showed especially potential in repressing the proliferation of P388 cell line with IC_50_ values of 0.36 μM (Mungmee et al., 2013). Therefore, its mechanism of action deserves to be further studied. The biphenyls **5**, **16–18**, **29, 31** and **37** also displayed promising cytotoxic effects, with IC_50_ values below 10 μM (Table 1). With regard to the bioactive qualities of other biphenyls, compound **3** is effective at inhibiting *Bacillus subtilis* with MIC values of 3.12 μg/mL (Cortez et al., 2002). Both compounds **44** and **45** were tested for antimicrobial activity. The MIC values for **45** were nearly half of those for **44** (Table 2). The results implied that the structural change from a 2-hydroxy-3-methyl-but-3-enyl side chain to a 3-hydroxymethyl-3-methyl-but-2-enyl side chain in **44** would have a positive influence on their antibacterial activity. Furthermore, the structure-activity relationship of compounds **16–18** based on the anti-tobacco mosaic virus activities (Table 3) indicated that the cyclization of the prenylated chain to form a six-membered heterocyclic ring might enhance the anti-TMV abilities. And biphenyl **50** is the most promising compound to resist tobacco mosaic virus with the inhibition rates of 31.1 ± 3.5% (Hu et al., 2016). Moreover, the compounds also performed well in antiviral test, with compound **52** exhibiting great potential against rotavirus with TI values of 17.65 (Gao et al., 2018).

Most of the biphenyls collected in this review have free hydroxyl groups, which may undergo metabolic deactivation by forming sulfates or glucuronides. This could be one of the reasons that some of the biphenyls in this review have moderate or no bioactivity. For example, compound **12** had no cytotoxicity against the P-388 cell line, whereas compound **13** with a protected C2 hydroxyl group had moderate cytotoxicity. Similarly, compound **37** with a tetrahydropyran ring had better cytotoxicity against P-388 and HT-29 compared to compound **36** with same core structure. To avoid the metabolic deactivation, the free hydroxyl group on the phenyl may be replaced by its bio-isosterism in the drug design. In addition, biphenyls are known for atropisomerism and have been exploited in drug discovery. It should be noted that compounds **42** and **60** with bulky groups next to C6 and C7 may have this structural feature.

Although biphenyl is a kind of compound with simple structure, it is worth to be studied because of its easy modification and diverse bioactivity. Several biphenyls from Clusiaceae do possess outstanding activity in multiple fields, such as compounds **6**, **50** and **52**. Evaluation of the action mechanism of these promising biphenyls should be noted and endeavored in the future.
